# Transcriptomic and metabolomic studies on the protective effect of molecular hydrogen against nuclear electromagnetic pulse-induced brain damage

**DOI:** 10.3389/fpubh.2023.1103022

**Published:** 2023-02-01

**Authors:** Long Ma, Shuo Tian, Hai-Ling Zhang, Jing-Yi Wang, Jia-Wen Wang, Hong-Li Yan, Xu-Guang Hu, Qi Shao, Jia-Ming Guo

**Affiliations:** ^1^Department of Radiation Medicine, College of Naval Medicine, Naval Medical University, Shanghai, China; ^2^Department of Clinical Laboratory, Beidaihe Rehabilitation and Recuperation Center of PLA, Qinhuangdao, China; ^3^Department of Biochemistry, College of Pharmacy, Naval Medical University, Shanghai, China; ^4^Department of Neurology, Shanghai Changhai Hospital, Naval Medical University, Shanghai, China; ^5^School of Basic Medicine, Naval Medical University, Shanghai, China; ^6^Incubation Base for Undergraduates' Innovation Practice, Department of Radiation Medicine, Faculty of Naval Medicine, Naval Medical University, Shanghai, China; ^7^Center of Reproductive Medicine, Shanghai Changhai Hospital, Naval Medical University, Shanghai, China; ^8^Department of Gastrointestinal Surgery, Shanghai Changhai Hospital, Naval Medical University, Shanghai, China; ^9^Institute of Neuroscience, Key Laboratory of Molecular Neurobiology of Ministry of Education and The Collaborative Innovation Center for Brain Science, Naval Medical University, Shanghai, China

**Keywords:** electromagnetic radiation, nuclear electromagnetic pulse, molecular hydrogen, radioprotectant, transcriptomics, metabolomics, glutathione

## Abstract

**Background:**

Excessive doses of electromagnetic radiation pose a negative impact on the central nervous system and lead to mental disorders. Molecular hydrogen can scavenge intracellular hydroxyl radicals, acting as an antioxidant, anti-apoptotic and anti-inflammatory agent. We seek to assess the capability of molecular hydrogen to ameliorate brain damage induced by electromagnetic radiation.

**Methods:**

NEMP (nuclear electromagnetic pulse), a subset of electromagnetic pulse with high voltage value that could cause severe brain injury, was applied to this study. Male wild-type rats were divided into four groups: the control group, the H_2_ (Molecular hydrogen) group, the NEMP group and the NEMP+H_2_ group. Rats in the H_2_ group and the NEMP+H_2_ group were fed with saturated hydrogen-rich water from 3 days before NEMP exposure (electromagnetic field intensity 400 kV/m, rising edge 20 ns and pulse width 200 ns) to the day of sacrifice. One day after exposure, animal behavior experiments were performed, and samples for transcriptomics and metabolomics analysis were collected. Seven days after exposure, histopathological experiments were conducted.

**Results:**

The data from the elevated plus maze and the open field test showed that NEMP exposure elicited anxiety-like behavior in rats, which could be alleviated by H_2_ treatment. Histopathological results manifested that NEMP exposure-induced injuries of the neurons in the hippocampus and amygdala could be attenuated by H_2_ treatment. Transcriptomic results revealed that NEMP exposure had a profound effect on microtubule structure in the brain. And the combined analysis of transcriptomics and metabolomics showed that H_2_ has a significant impact on the neuroactive ligand-receptor interaction, synaptic vesicle cycle and synapse etc. Moreover, it was indicated that the glutathione metabolic pathway played a vital role in the NEMP exposure-induced damage and the protective activity of H_2_.

**Conclusions:**

H_2_ is identified as a potent agent against NEMP exposure-induced brain damage and has the potential to be a promising electromagnetic radiation protectant.

## Background

In recent decades, with the rapid development of communication science, electromagnetic-based technology has been widely used in various fields of human productive activity and daily life. Huge amounts of researches have reported that electromagnetic radiation (EMR) has become an important pollution source in modern civilization that should not be ignored (1). Due to the prolonged use of mobile phones, the physical proximity of mobile phones to the brain in communication, and the substantial increase in the electromagnetic intensity of mobile phones in communication, the brain is exposed to more EMR than other organs. Epidemiological and laboratory studies have demonstrated that exposure to electromagnetic fields can result in insomnia, headache, tinnitus, fatigue, cognitive impairment, paresthesias, dizziness and anxiety (2–4). Furthermore, the International Agency for Research on Cancer (IARC) classified radiofrequency electromagnetic fields (30 kHz−300 GHz) as a potential human carcinogen (Group 2B) in 2011 (5). Thus, the detrimental effect of EMR on human brains deserves more attention.

Electromagnetic pulse (EMP) is a special type of electromagnetic field, and its waveform is a short, high-voltage pulse wave with extremely fast rise times and a wide bandwidth from 0 Hz to 1.5 GHz. The central nervous system is prone to EMP exposure-induced injuries. Exposure to EMP increases microvascular permeability in the brain of the rat, which then leads to an increase in albumin penetration into the brain (6). NEMP is a subset of EMPs that were first observed at the instant of a nuclear explosion. It has the characteristics of strong field, short action time, wide spectrum and wide action range. NEMP can paralyze electronic equipment and communication equipment in a short time, demonstrating considerable military application value. Compared with other EMPs, NEMP has higher voltage values and can cause more severe brain damage. At present, the research on NEMP mainly focuses on its destructive effect on electronic equipment and shielding devices, whereas its potential threat to the health of organisms is rarely reported.

Considering the negative effects of EMR exposure on the human body, especially the central nervous system, it is urgent to develop safe and effective EMR protectant. Molecular hydrogen, as a new type of antioxidant that can selectively scavenge hydroxyl radicals, is non-toxic and can easily penetrate biological membranes. Studies have shown that molecular hydrogen can play a protective role in various conditions such as ischemia/reperfusion injury, organ transplantation, metabolic syndrome, inflammation and acute irradiation disease (7–9). It has also been reported that H_2_ could alleviate neurological disorders caused by oxidative stress and neuroinflammation (10). In the pre-experimental phase, we screened potential biological protective agents for central nervous system damage induced by EMR. Intriguingly, H_2_ exhibits a dramatic protective effect (11).

In this study, we investigated the effects of NEMP exposure on the rat brain and the protective role of molecular hydrogen. Furthermore, we used transcriptomics and metabolomics to analyze the underlying mechanism.

## Methods

### Animals and grouping

Male wild-type Sprague-Dawley (SD) rats (8 weeks old, weighing 200–220 g) were received from the Shanghai Laboratory Animal Center of Chinese Academy of Science and maintained at 23–25°C with a 12 h light/dark cycle. Before the experiment, rats were housed for 1 week to adapt to the new environment. All living conditions and protocols were approved by the Naval Medical University Animal Welfare and Ethics Committee in accordance with the Guide for Care and Use of Laboratory Animals published by the US NIH (publication No. 96-01). These rats were randomly divided into four groups: the control group, the H_2_ group, the NEMP group, and the NEMP+H_2_ group.

### Molecular hydrogen treatment and NEMP exposure

**Figure 1:**
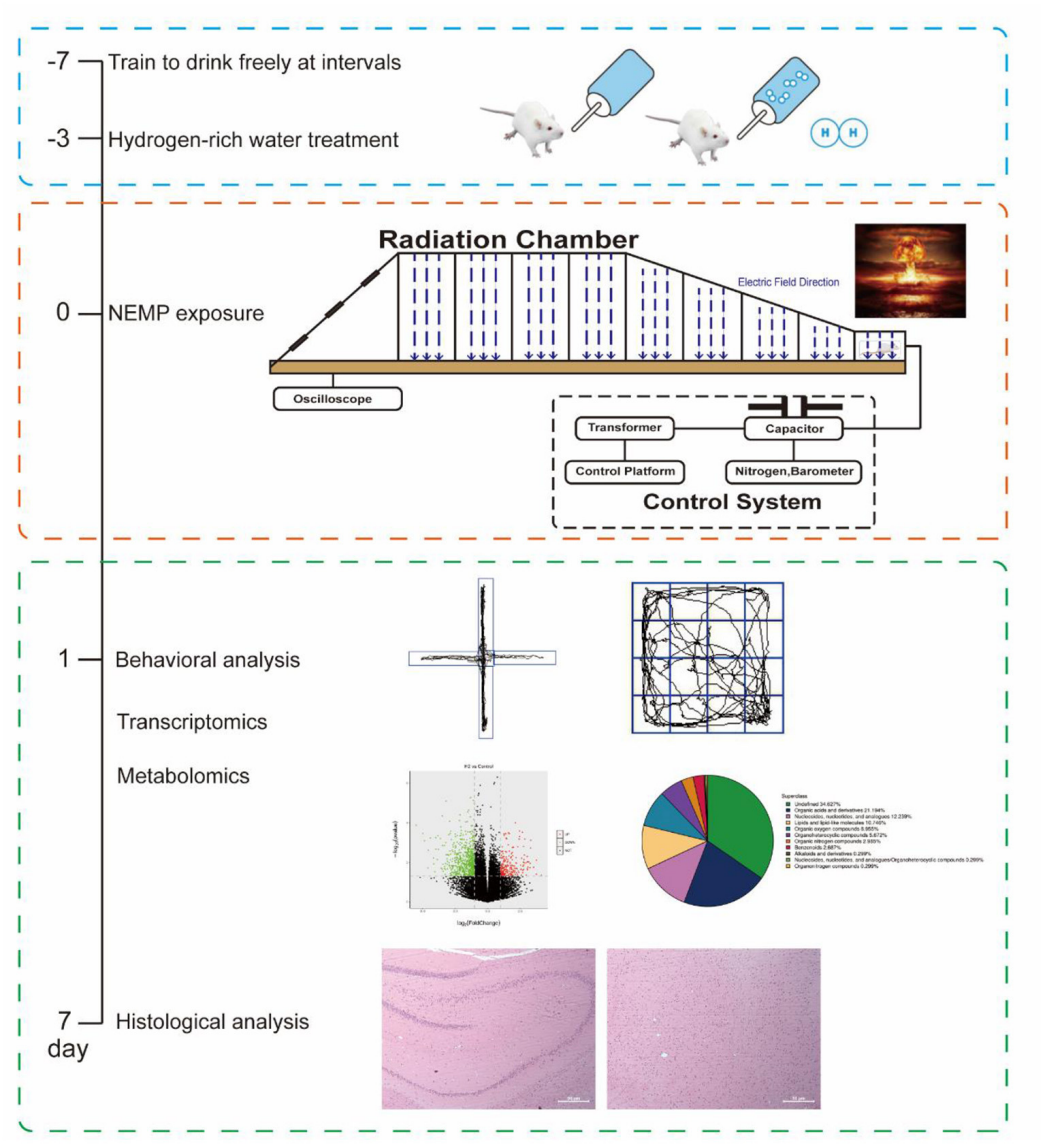
Schematic workflow of the research process and some of the representative images from the experiments.

**Figure 2:**
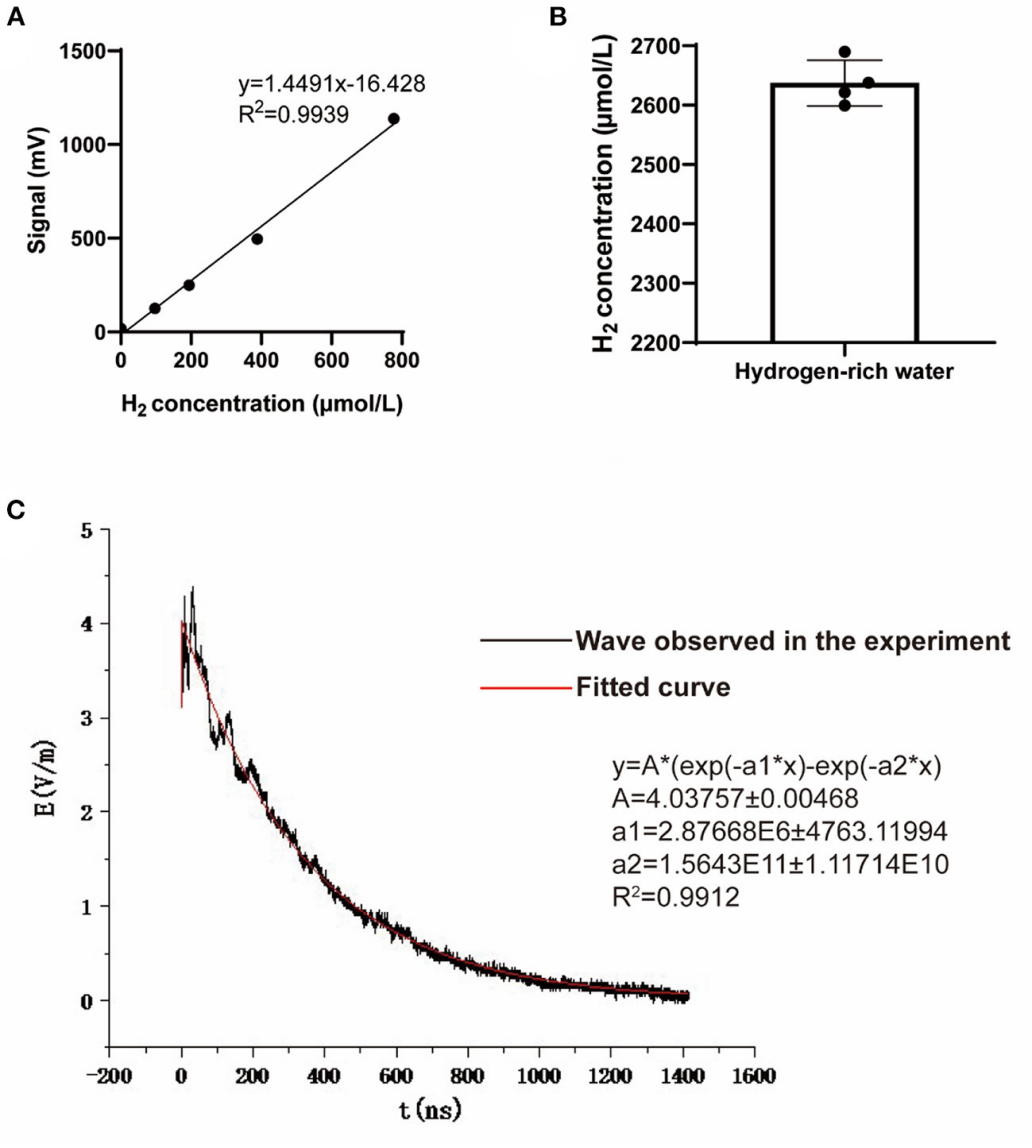
Hydrogen concentration of the hydrogen-rich water and the waveform of the NEMP. **(A)** The standard curve used to detect hydrogen concentration in hydrogen-rich water. **(B)** The hydrogen concentration of the hydrogen-rich water used in this study. *N* = 4. **(C)** The waveform and paraments of the NEMP used in this study.

To ensure the stability and homogeneity of the hydrogen water treatment, we adopted the freshly produced hydrogen-rich water (HRW), which was prepared with an automatic “hydrogen water generator” (High-concentration HRW generator, GL-110-PW, SHENZHEN SMART WATER TECH. LTD., Guangdong, China) before use each time, to feed the rats as we previously conducted (11). In brief, the tank of the generator was filled with the autoclaved drinking water and then subjected to the hydrogen generation process. Ten minutes later, the HRW was ready for both the concentration tests with a microelectrode (Unisense, Aarhus N, Denmark) and the followed animal treatments. To train rats to drink hydrogen-rich water, the rats were fed with intermittent drinking water for 1 week, and water was only given at 8–10, 12–14, and 16–18 o 'clock every day. The H_2_ group and the NEMP+H_2_ group were given saturated hydrogen-rich water, while the control group and NEMP group were given ordinary autoclaved drinking water. The water supply time is 8–10, 12–14, 16–18 o 'clock every day (Figure 1). The molecular hydrogen treatment lasted from 3 days before NEMP exposure to the day of sacrifice (Figures 2A, B).

The NEMP exposure system used in this study was developed and tested at the Department of Biophysics, East China Normal University, Shanghai, China. The system is composed of a voltage control system (control platform, booster transformer, pulse capacitor), an air pressure control system (high-purity nitrogen gas, air valve, barometer), a radiation chamber and a pulse detection system (voltage divider, coaxial cable, oscilloscope). Before radiation exposure, the high purity nitrogen gas in the pulse capacitor is controlled by air pressure control valve and air pressure meter, and the voltage between two plates of capacitance is increased by booster transformer. When the voltage reaches a certain value, the nitrogen gas is broken down, and an electrical pulse is generated between the metal lead plates connected with the capacitance, which propagates along the radiation chamber to the end. NEMP is attenuated by resistance to prevent the formation of reflection. In this experiment, an oscilloscope connected to the sampling resistor on the irradiation chamber was used to detect the waveform (rising edge 20 ns and pulse width 200 ns, Figure 2C). The whole body of each rat was exposed to 200 pulses with an electromagnetic field intensity of 400 kV/m, and their rectal temperature was kept to be monitored and found to be elevated less than 0.3°C by the exposure, in accordance to that of previous reports (12, 13).

### Elevated plus maze

EPM was conducted to evaluate the anxiety-like behaviors based on the rodents' innate aversion to open and elevated areas. The EPM apparatus consists of two closed arms and two open arms, which are made of acryl material and placed at a height of 50 cm above the floor. Rats tend to move around in closed arms due to their penchant for darkness, but they will be active in the open arm again out of curiosity and inquisitiveness. When confronted with novel stimuli, rats develop both the urge to explore and fear, which results in conflicting behaviors of exploration and avoidance, leading to anxiety. EPM is considered a classic experiment for assessing anxiety and stress research. At the beginning of each test, the rats were placed in a central zone facing the open arm and allowed to explore freely for 10 min. The movements of the animals were captured with a digital camera and analyzed by software (*Mobile Data Information Technology Co., Ltd., Shanghai, China*). The total entries into the arms, the percentage of distance traveled in the open arms, and the percentage of open arms residence time were analyzed. The maze was cleaned with an alcohol solution before each test. Each group contained 10 rats.

### Open field test

OFT is typically used to evaluate the autonomous behavior, exploratory behavior and tension of experimental animals in a novel and different environment. The frequency and duration of certain behaviors in the novel environment were used to respond to the rats' autonomous and exploratory behaviors in the unfamiliar environment, and the number of urine and stool was used to respond to their nervousness. The OFT apparatus is composed of a black wooden box (100 × 100 × 35 cm) and a black floor. Rats were placed in the center of the apparatus under dim lighting and the total distance traveled for 6 min was monitored by a computer-controlled tracking system (*Mobile Data Information Technology Co., Ltd., Shanghai, China*). The total distance, distance traveled in the center area, and time spent in the central area were measured to evaluate the level of anxiety. After each individual test, the apparatus was cleaned with an alcohol solution. Each group contained 10 rats.

### Hematoxylin and eosin and Nissl staining

After finishing the animal behavior experiments, rats were sacrificed 7 days after NEMP exposure to conduct the histological examinations. During the animal termination procedure, rats were anesthetized for tissue perfusion and then euthanized for tissue collection. To relieve the pain induced by the anesthetic's injection, rats were firstly subjected to the inhalation of isoflurane (5% in oxygen) to induce a quick unconsciousness and then the followed intraperitoneal injection of sodium pentobarbital (9.1 mg/ml, 80 mg/kg). Not until was there no responses to the rat feet pinching, did the heart perfusion (injection from the left ventricle and liquid effusing via the right atrium) was initiated by normal saline (0.9%, 250ml, 40ml/min) and paraformaldehyde (4%, 500ml, 20ml/min), sequentially. When the perfusion process was finished, brain tissue was harvested for the followed experiments such as H&E and Nissl staining. Considering the homogeneity between the two encephalon hemispheres in terms of the damage to the brains by NEMP exposure, we only carried out the histopathological study in right hemispheric brain. The right hemispheric brain of each rat was dissected, fixed with 4% paraformaldehyde for at least 24 h, and then dehydrated with a graded ethanol series. Subsequently, the specimen was embedded in paraffin, cut into 4 μm thick slices. The slices were stained in H&E staining working solution and Nissl staining working solution, respectively. Then the slices were observed and photographed under an optical microscope. Each group contained four rats.

### Transcriptomics analysis

After homogenization, total RNA from rat brain was extracted and purified using the RNeasy mini kit (Qiagen, Hilden, Germany). Next, cDNA libraries were built using the TruSeq^®^ Stranded Total RNA Sample Preparation Kit (Illumina, San Diego, California, USA) according to the manufacturer's instructions. Subsequently, sequencing was performed on the Illumina HiSeq 2000 System (Illumina, San Diego, California, USA). Differentially expressed genes (DEG) were selected using the following filter criteria: *p* < 0.05 and fold change >2. Each group contained eight rats.

### Metabolomics analysis

The samples were analyzed using ultra-high performance liquid chromatography (1,290 Infinity LC, Agilent Technologies, Santa Clara, California, USA) coupled to a quadrupole time-of-flight system (AB Sciex TripleTOF 6600, SCIEX, Redwood City, California, USA). Metabolites were identified by comparing their mass spectra with an in-house database established using available authentic standards (Shanghai Applied Protein Technology Co. Ltd, Shanghai, China). Univariate analysis was performed to screen out metabolites that met the criteria of foldchange>1.5 or <0.67 and *p* < 0.05 (Supplementary Figure 1). Subsequently, a principal component analysis (PCA), partial least-squares discriminant analysis (PLS-DA), PLS-DA permutation test, orthogonal partial least-squares discriminant analysis (OPLS-DA), and OPLS-DA permutation test were performed (Supplementary Figures 2–7). The OPLS-DA model variable importance in the projection (VIP) values of each metabolite were calculated. Significant differential metabolites (DM) were selected using the following filter criteria: *p* < 0.05 and VIP value >1. Each group contained eight rats.

### Combined analysis of transcriptomics analysis and metabolomics analysis

All differentially expressed genes and metabolites were mapped to Kyoto Encyclopedia of Genes and Genomes (KEGG, http://www.kegg.jp/) pathways according to the online and enrichment analysis was performed. Subsequently, Venn diagrams and histograms were plotted in combination with KEGG annotations and enrichment results of the two omics.

### Statistical analysis

All data were presented as mean ± SD and statistical analysis was performed using SPSS 22.0 software (SPSS Inc., Chicago, USA). GraphPad Prism 8 Software (GraphPad Software Inc., California, USA) was used to make the graphs. Statistical significance between the two groups of data that were in accordance with the normal distribution was determined by the student's *T*-test. Differences were considered statistically significant when the *p* < 0.05.

## Results

### Anxiety-like behaviors

**Figure 3:**
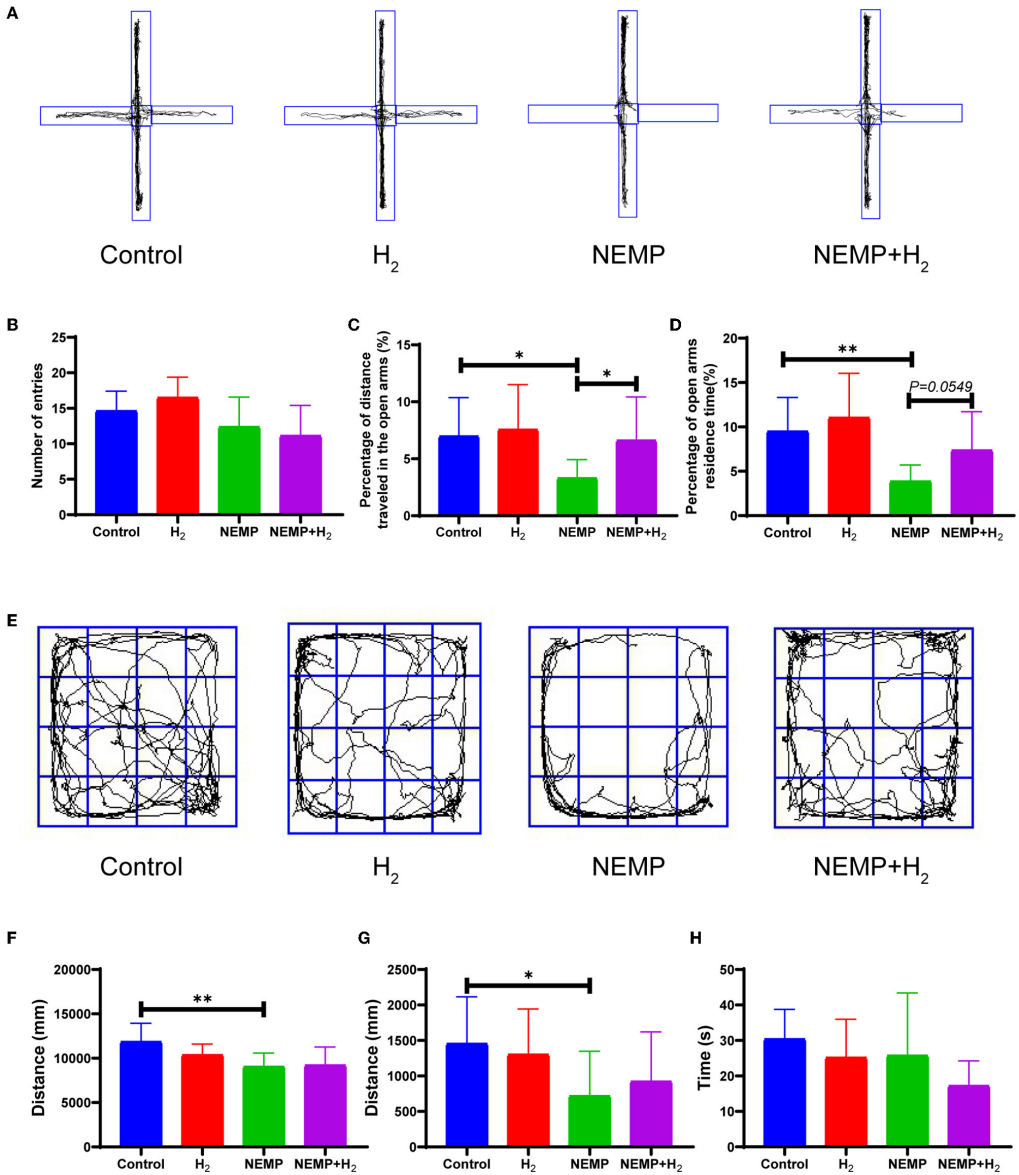
Effects of EMP exposure and H_2_ administration on anxiety-like behavior. **(A)** Representative movement track diagrams of rats in EPM. **(B)** Total number of entries into the arms in EPM. **(C)** Percentage of distance traveled in the open arms in EPM. **(D)** Percentage of open arms residence time in EPM. **(E)** Representative movement track pictures of rats in OFT. **(F)** Total distance traveled in OFT. **(G)** Distance traveled in the center area in OFT. **(H)** Time spent in the central area in OFT. *N* = 10. All data are presented as mean ± SD. *p < 0.05, **p < 0.01.

The anxiety-like behaviors of rats were evaluated by EPM and OFT. The EPM results showed no significant difference in the total number of entries into the arms among the four groups, indicating no change in locomotor activity in rats after NEMP exposure and/or H_2_ treatment (Figure 3B). The percentage of distance traveled in the open arms and the percentage of open arms residence time decreased significantly in the NEMP group, compared with the control group (Figures 3A, C, D). Interestingly, the percentage of distance traveled in the open arms and the percentage of open arms residence time increased significantly in the NEMP+H_2_ group, compared with the NEMP group (Figures 3A, C, D). These results suggested that NEMP exposure could increase the animals' anxiety-like behavior, which were attenuated by H_2_.

In addition, we found a significant difference in the total distance and the distance traveled in the center area in OFT between the control group and the NEMP group (Figures 3E–H), indicating that NEMP exposure could induce an anxiety-like behavior. However, the NEMP+H_2_ group showed a tendency of traveling more distance in the center area in OFT (Figure 3G), suggesting that the anxiety-like behavior was improved mildly by H_2_.

### Histological changes

**Figure 4:**
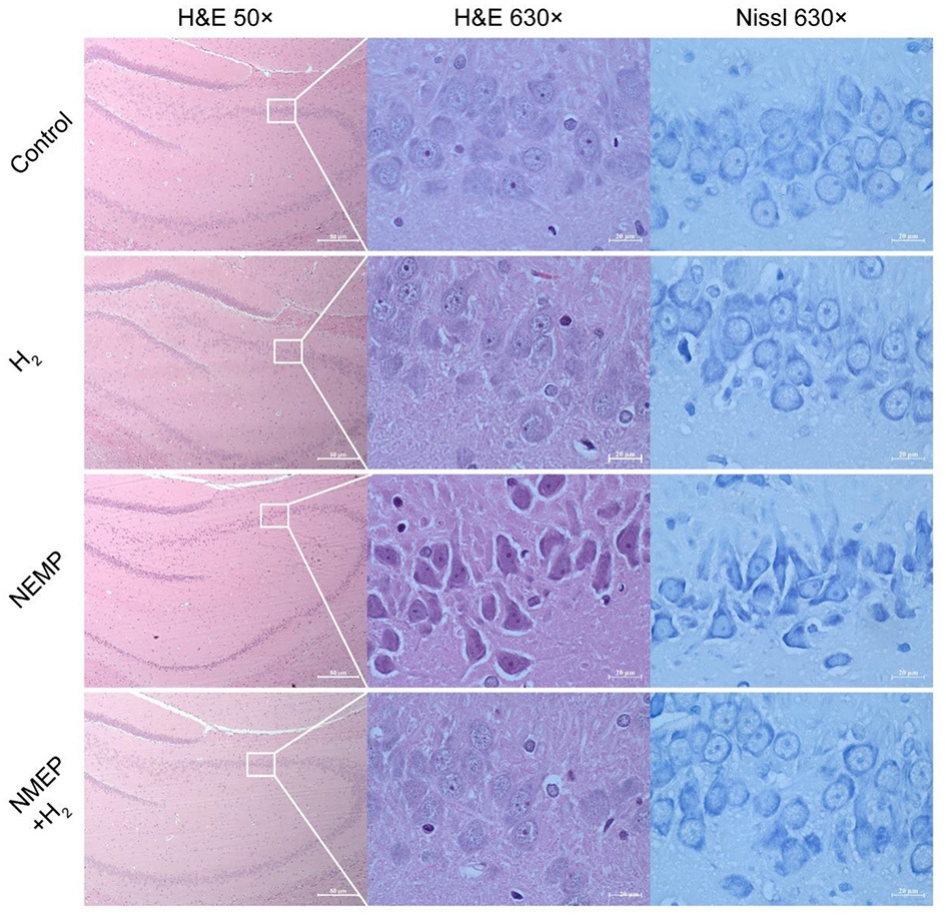
Representative micrographs of the hippocampus each group (H&E and Nissl staining). *N* = 4.

In the present study, the hippocampus and amygdala were the objectives of light microscopic analysis. As one of the principal tissue stains used in histopathology, we used H&E to study the pathological changes to the nucleus and the cytoplasmic inclusions of hippocampal and amygdala neurons. As demonstrated by H&E staining and Nissl staining in Figure 4, NEMP exposure led to significant pathological changes in rat whole hippocampus and amygdala neurons during the delayed phase after exposure to NEMP (7 days after exposure): cell swelling, pronounced cell gaps, morphological changes (from round and oval to triangular), deep cell staining, and blurring of the nucleus and cytoplasm. Of note, the pathological changes of hippocampal neurons in the brain tissue of rats in the NEMP+H_2_ group were significantly smaller than those in the NEMP group, and the proportion of pathological neuronal cells was significantly lower. Similarly, more abnormal neuron cells were found in the amygdala of rats in the NEMP group, and H_2_ significantly reduced the proportion of abnormal cells (Supplementary Figure 8). According to our observations at low magnification, most neurons in the whole hippocampal and amygdala underwent similar changes after NEMP exposure, which were counteracted by H_2_. There was no significant difference in the morphology of glial cells and blood vessels among these four groups, suggesting that neurons are more sensitive to NEMP exposure.

### Transcriptomics analysis

Heatmap and volcano plots were used to show the changes in gene expression profiles (Supplementary Figures 9, 10). The results showed that there were 178 up-regulated and 502 down-regulated DEGs in the H_2_ group, 77 up-regulated DEGs and 166 down-regulated DEGs in the NEMP group compared with the control group. Meanwhile, H_2_ upregulated the expression of 209 genes and downregulated the expression of 403 genes in rat brain after NEMP exposure.

**Figure 5:**
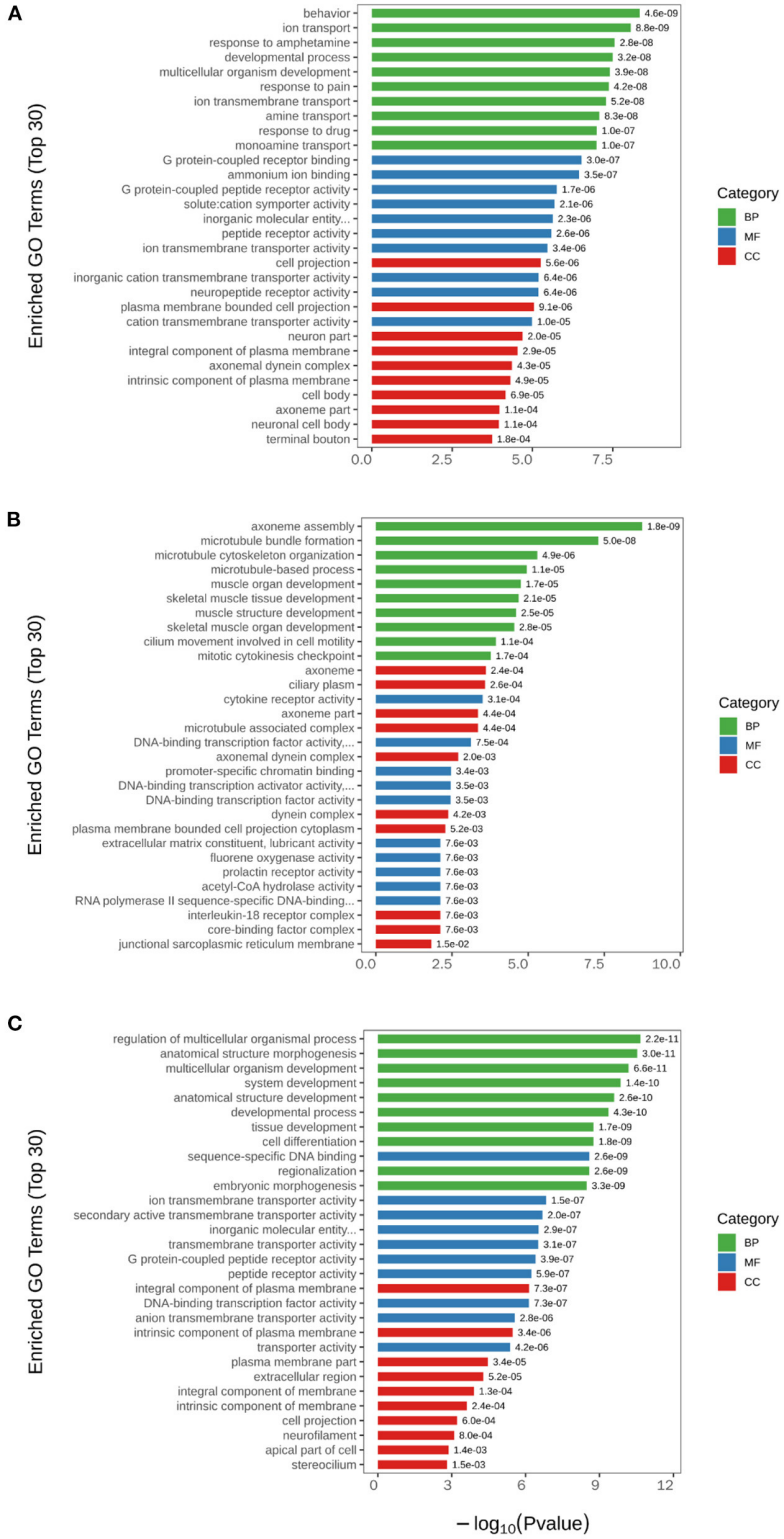
GO analysis of significant DEGs. **(A)** H_2_ group vs. control group. **(B)** NEMP group vs. control group. **(C)** NEMP+H_2_ group vs. NEMP group. The vertical coordinates are top 30 enriched GO terms. The horizontal coordinate is –log10 (*P*-value). The green columns represent BP terms; the blue columns represent MF terms; the red columns represent CC terms. *N* = 8.

Subsequently, GO pathway enrichment analysis was performed for DEGs in each group, and the top 30 GO terms with statistical significance are shown in Figure 5. The results showed that among the three main terms of GO [biological process (BP), molecular function (MF) and cellular component (CC)], BP was obviously enriched (green column). The BP terms induced by H_2_ treatment alone including behavior, ion transport, response to amphetamine, developmental process, etc. The BP terms caused by NEMP exposure alone including axoneme assembly, microtubule bundle formation, microtubule cytoskeleton organization, microtubule-based process, etc. The BP terms altered in NEMP+H_2_ group compared with the NEMP group contained regulation of multicellular organismal process, anatomical structure morphogenesis, multicellular organism development, system development, etc.

### Metabolomics analysis

Differential metabolites are listed in Table 1 and Supplementary Table 1. Compared to the control group, 6 metabolites were down-regulated (L-Fucose, 2-Methylglutaric acid, cis-Aconitate, Hypoxanthine, Uracil, and L-Glutamate) and 4 metabolites (2-Amino-2-methyl-1,3-propanediol, 1-Palmitoyl-2-oleoyl-sn-glycero-3-phosphoethanolamine, Sphingosine, and Pantetheine) were up-regulated in the H_2_ group. Compared to the control group, 16 metabolites were down-regulated (Fosfomycin, Allocystathionine, Adenine, L-Histidine, Argininosuccinic acid, Isopentenyladenosine, Glycerophosphocholine, Cyclohexylamine, L-Fucose, 2-Methylglutaric acid, L-Leucine, Uracil, Pantetheine, L-Phenylalanine, Dopamine, and D-Proline) and 3 metabolites (Heptadecanoic acid, Sphingosine, 1-Palmitoyl-2-hydroxy-sn-glycero-3-phosphoethanolamine) were up-regulated in the H_2_ group. Only one metabolite D-Arabinono-1,4-lactone was elevated in the NEMP+H_2_ group compared to the NEMP group.

**Table 1:** Differential metabolites between H_2_ vs. control, NEMP vs. control, and NEMP+H_2_ vs. NEMP.

**H**_**2**_ **vs. control**				**NEMP vs. control**				**NEMP**+**H**_**2**_ **vs. NEMP**			
**Name**	**VIP** ^*^	**Foldchange**	***p***	**Name**	**VIP**	**Foldchange**	***p***	**Name**	**VIP**	**Foldchange**	***p***
L-Fucose	1.2070	0.5062	0.0329	Fosfomycin	2.3762	0.0388	0.0122	D-Arabinono-1,4-lactone	1.2846	1.6026	0.0192
2-Methylglutaric acid	1.5240	0.6909	0.0485	Allocystathionine	1.1706	0.2787	0.0126				
cis-Aconitate	1.8308	0.7048	0.0277	Adenine	7.6889	0.4490	0.0111				
Hypoxanthine	27.0826	0.7337	0.0416	L-Histidine	4.1197	0.4556	0.0027				
Uracil	9.1489	0.7463	0.0271	Argininosuccinic acid	1.6033	0.4591	0.0003				
L-Glutamate	4.5553	0.8517	0.0438	Isopentenyladenosine	1.7778	0.4844	0.0111				
2-Amino-2-methyl-1,3-propanediol	1.0485	1.3249	0.0471	Glycerophosphocholine	1.3364	0.5161	0.0401				
1-Palmitoyl-2-oleoyl-sn-glycero-3-phosphoethanolamine	1.5903	1.3463	0.0477	Cyclohexylamine	3.5729	0.5335	0.0058				
Sphingosine	2.8488	1.3961	0.0468	L-Fucose	1.4834	0.5899	0.0298				
Pantetheine	4.4592	1.6149	0.0396	2-Methylglutaric acid	1.3983	0.6111	0.0299				
				L-Leucine	4.3383	0.6584	0.0088				
				Uracil	9.4206	0.6638	0.0176				
				Pantetheine	2.9790	0.6981	0.0485				
				L-Phenylalanine	1.3551	0.7083	0.0071				
				Dopamine	1.3297	0.7289	0.0093				
				D-Proline	5.0925	0.7489	0.0095				
				Heptadecanoic acid	1.5842	1.4100	0.0213				
				Sphingosine	2.2820	1.6644	0.0474				
				1-Palmitoyl-2-hydroxy-sn-glycero-3-phosphoethanolamine	2.8960	2.1258	0.0025				

* VIP, variable importance in the projection.

### Combined transcriptomics and metabolomics KEGG pathway analysis

Subsequently, a combined KEGG pathway analysis of DEGs and differential metabolite was performed to obtain more detailed information on the effects of NEMP exposure and H_2_ treatment on metabolic pathways in rat brain tissue. VENN plot analysis was done for the KEGG pathway enriched by transcriptomics and metabolomics, and their intersections were further enriched (due to the small number of differential metabolites in some of the comparison groups, the KEGG pathway of all identified metabolites was chosen here).

**Figure 6:**
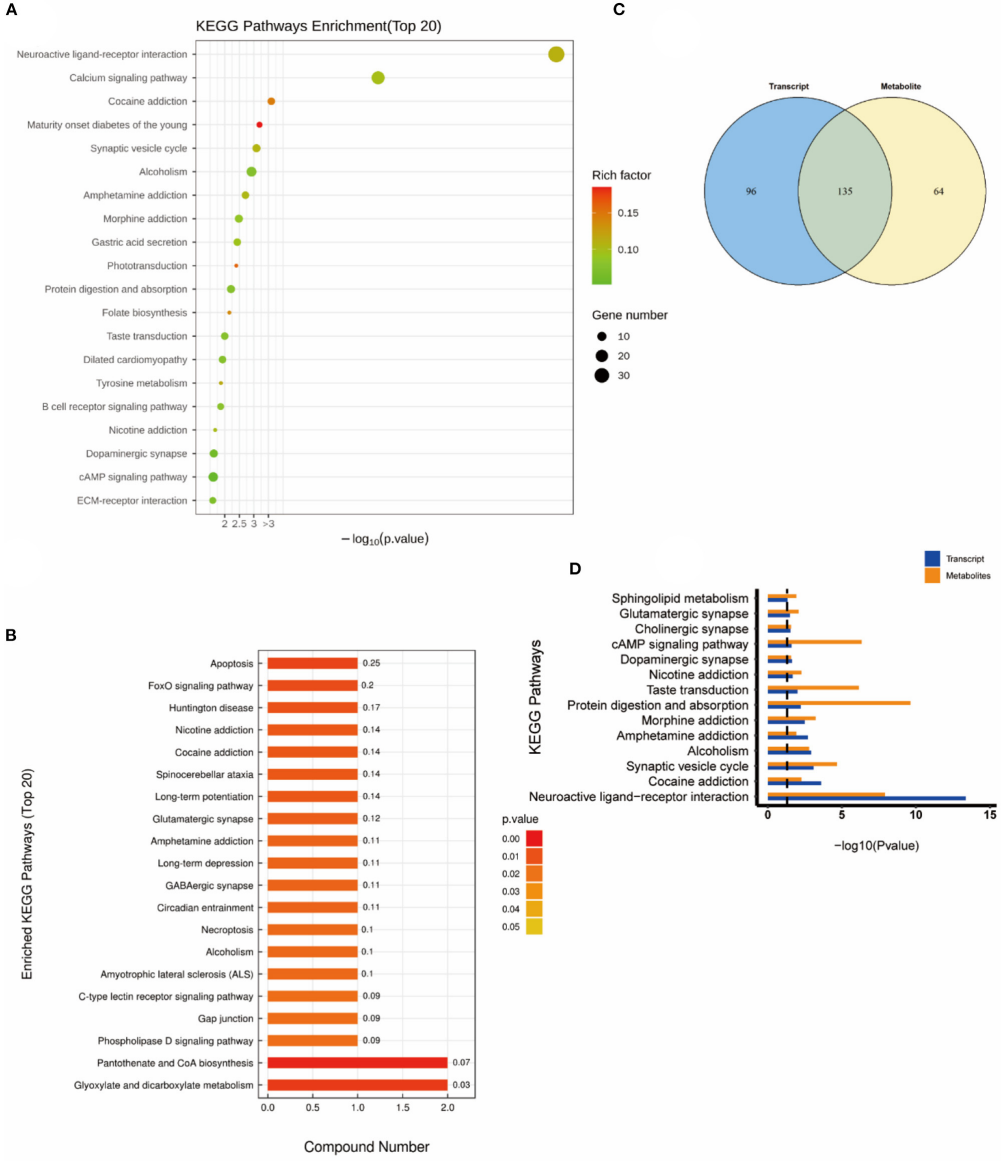
KEGG pathway analyses of DEGs and DMs identified by comparing the H_2_ group and the control group. **(A)** KEGG pathway analyses of DEGs. **(B)** KEGG pathway analyses of DMs. **(C)** Venn diagram of the KEGG pathway involving DEGs and DMs. **(D)** Enrichment analysis of KEGG pathway between DEGs and DMs. *N* = 8.

**Figure 7:**
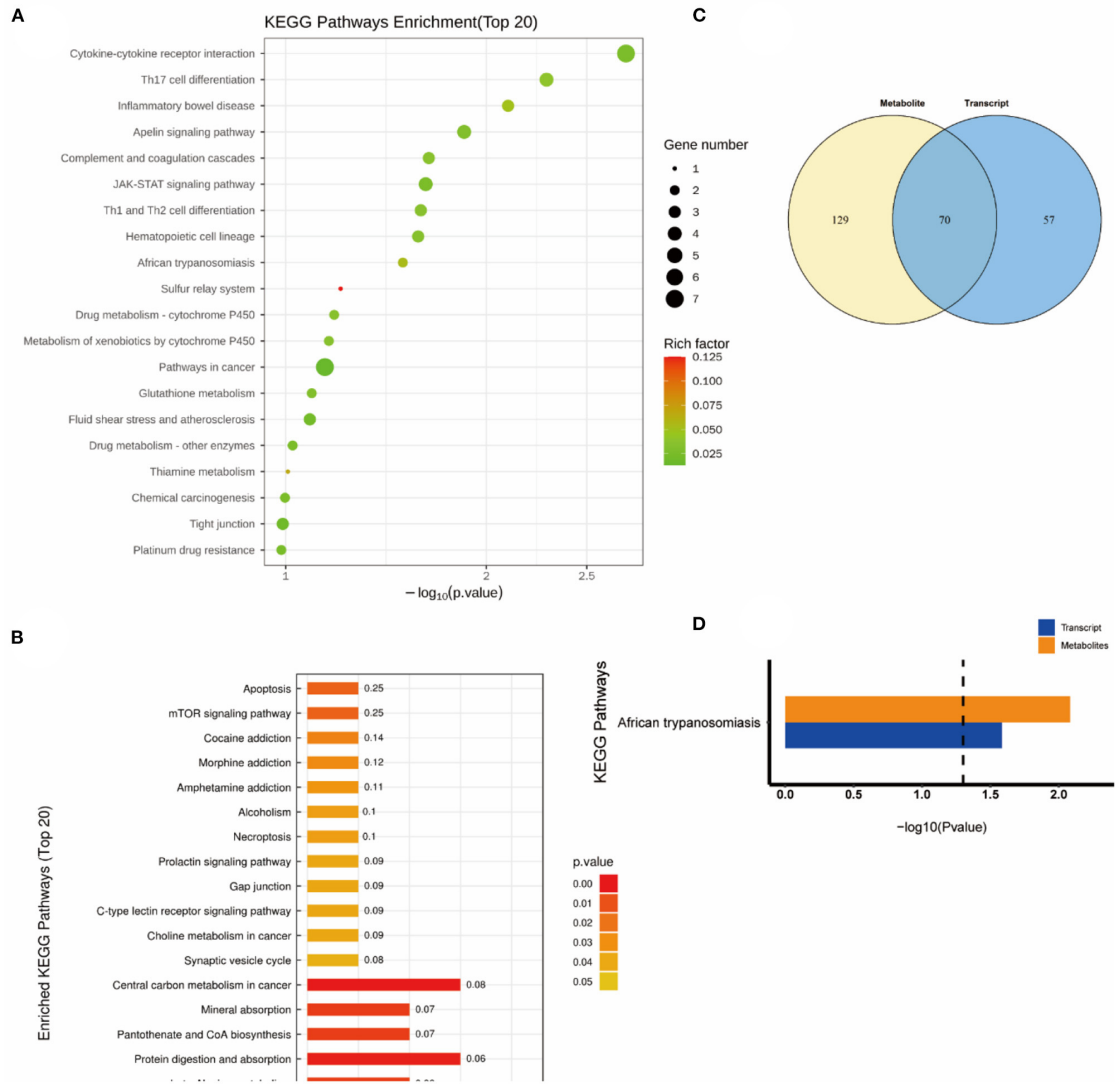
KEGG pathway analyses of DEGs and DMs identified by comparing the NEMP group and the control group. **(A)** KEGG pathway analyses of DEGs. **(B)** KEGG pathway analyses of DMs. **(C)** Venn diagram of the KEGG pathway involving DEGs and DMs. **(D)** Enrichment analysis of KEGG pathway between DEGs and DMs. *N* = 8.

**Figure 8:**
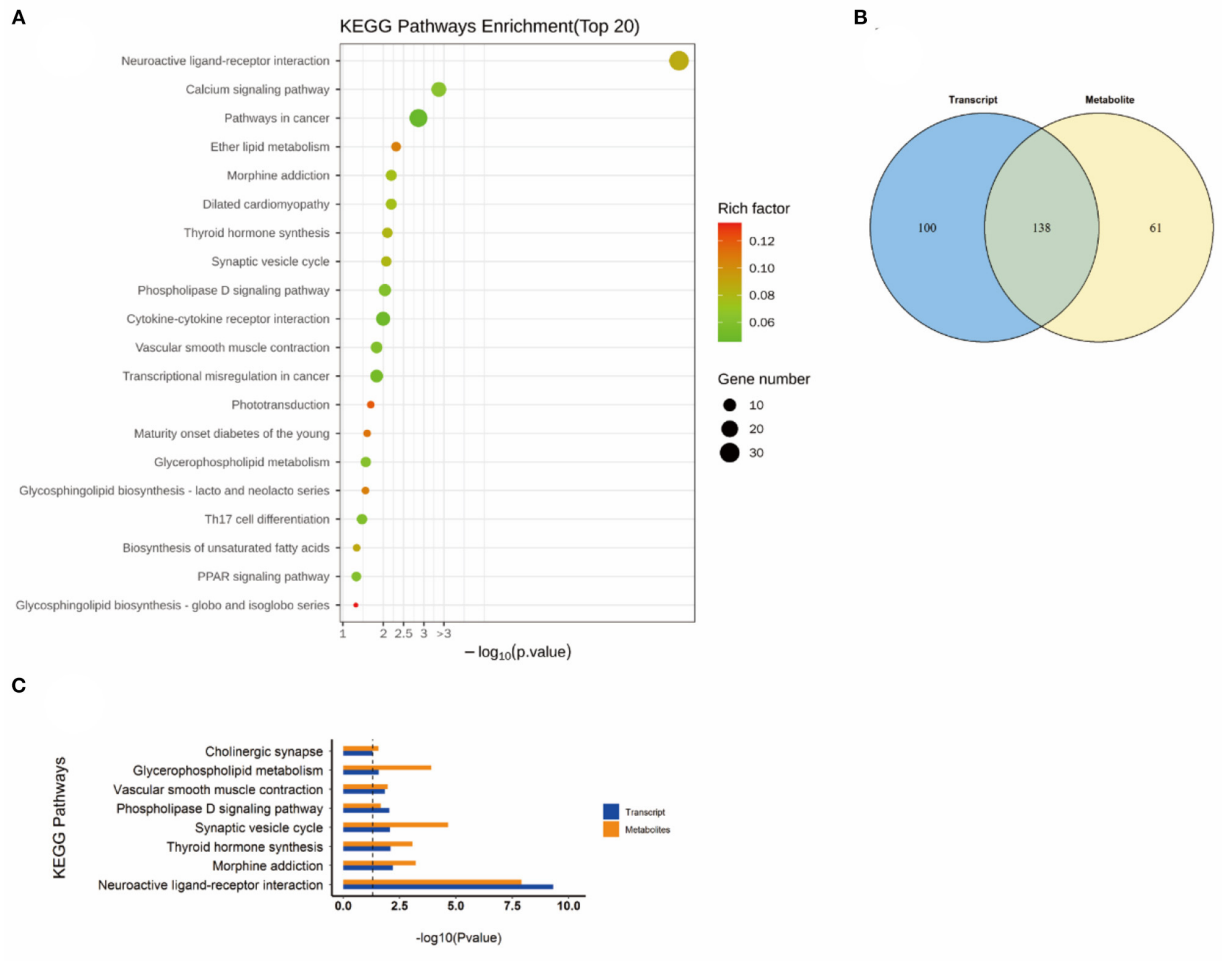
KEGG pathway analyses of DEGs and DMs identified by comparing the NEMP+H_2_ group and the NEMP group. **(A)** KEGG pathway analyses of DEGs. **(B)** Venn diagram of the KEGG pathway involving DEGs and DMs. **(C)** Enrichment analysis of KEGG pathway between DEGs and DMs. *N* = 8.

As shown in Figure 6, the altered KEGG pathways induced by H_2_ treatment alone mainly included neuroactive ligand-receptor interaction, synaptic vesicle cycle, (dopaminergic, cholinergic, and glutamatergic) synapse, cAMP signaling pathway, drug addiction, etc. Compared with the control group, only one metabolic pathway was enriched in the NEMP group, and it was almost irrelevant (Figure 7). The KEGG pathways altered in NEMP+H_2_ group compared with the NEMP group contained neuroactive ligand-receptor interaction, synaptic vesicle cycle, phospholipase D signaling pathway, vascular smooth muscle contraction, glycerophospholipid metabolism, cholinergic synapse, etc. (Figure 8).

**Figure 9:**
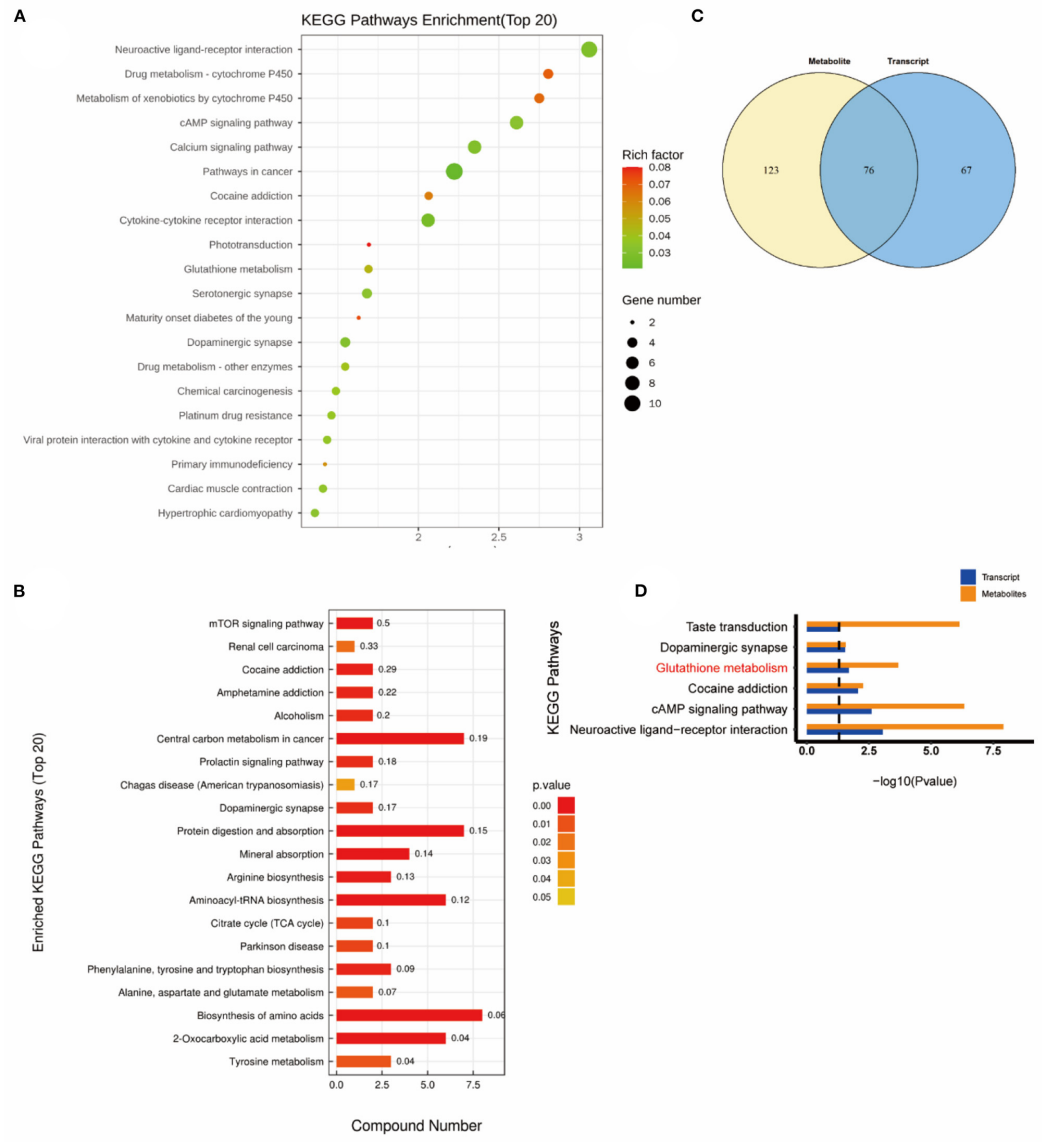
KEGG pathway analyses of DEGs and DMs identified by comparing the H_2_ group and the NEMP group. **(A)** KEGG pathway analyses of DEGs. **(B)** Venn diagram of the KEGG pathway involving DEGs and DMs. **(C)** Enrichment analysis of KEGG Pathway between DEGs and DMs. **(D)** Enrichment analysis of KEGG pathway between DEGs and DMs. *N* = 8.

Interestingly, the glutathione metabolic pathway was significantly enriched when comparing the KEGG pathway between the H_2_ group and the NEMP group (Figure 9).

### Glutathione metabolic pathway analysis

**Figure 10:**
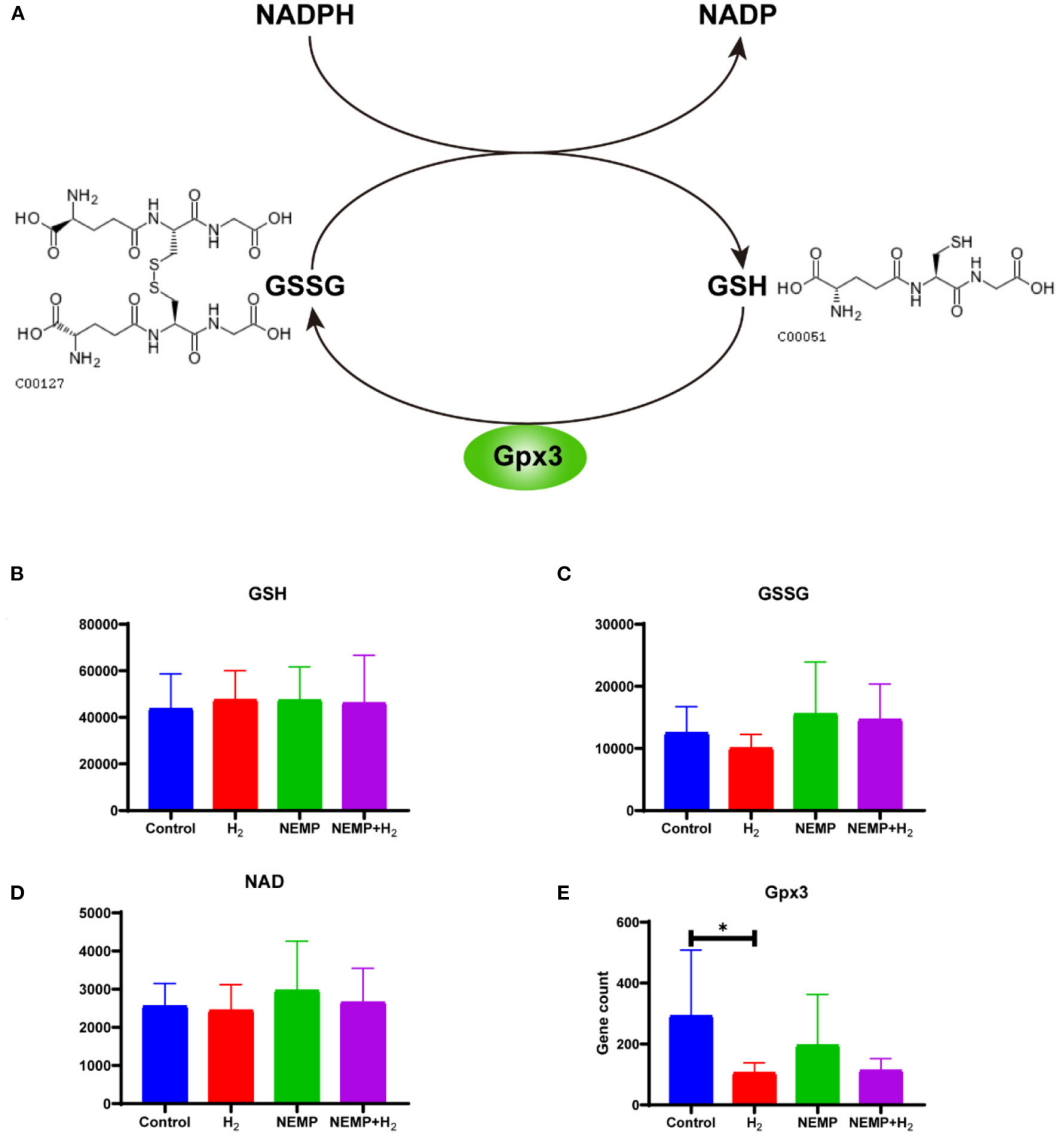
Effects of NEMP exposure and molecular hydrogen treatment on glutathione metabolic pathways. **(A)** Diagram of the glutathione cycle. **(B)** GSH levels. **(C)** GSSG. **(D)** NADP levels. **(E)** Gpx3 gene counts. *N* = 8. ^*^p < 0.05.

Glutathione is the most important active metabolite with antioxidant effect that is naturally present in cells. Glutathione is available in both reduced forms (glutathione, GSH) and oxidized forms (glutathione disulfide, GSSG), and glutathione reductase catalyzes the interconversion between the two forms, and the coenzyme of this enzyme also provides NADPH for pentose phosphate bypass metabolism (Figure 10).

The results of this study showed that there was no difference in GSH levels among the four groups. However, it can be observed that: GSSG was reduced after molecular hydrogen treatment alone, GSSG was elevated after NEMP exposure, and GSSG was reduced in the NEMP+H_2_ group compared to the NEMP group (*n* = 8, no differences were not statistically significant due to large intra-group dispersion). Similar trends were also observed in NADP levels. Meanwhile, Gpx3, a GSH oxidase, was significantly reduced after hydrogen treatment alone (*P* < 0.05).

## Discussion

Increasing number of studies have demonstrated that EMR can have a negative impact on human, of which the brain is one of the most vulnerable organs (1, 14–16). It has been reported in a literature that the damage caused by EMR is related to mitochondrial damage and redox imbalance (17). Meanwhile, supplementation with some antioxidants or anti-free radicals, such as vitamin E and melatonin, has exhibited a protective role in specific population from EMR (18). Molecular hydrogen, as a free radical scavenger, also demonstrates antioxidant, anti-apoptotic and ionizing radiation damage-protective effects. In the current study, we establish a protective role of molecular hydrogen against NEMP exposure-induced damage by conducting animal behavior experiments and histopathological experiments. Mechanistically, transcriptomics and metabolomics are adopted to decode potential protective mechanisms.

Epidemiological studies provided abundant evidence that electromagnetic fields from cell phones, cell phone base stations and WIFI devices all had similar undesirable neuropsychiatric effects in humans, and some of these studies had shown a clear dose-response relationship between electromagnetic intensity and health problems (19). The most common disorders or diseases induced by electromagnetic fields include insomnia, headache, depression, tiredness, insensitivity, inattention, memory changes, dizziness, irritability, loss of appetite, anxiety, nausea, skin burning (or tingling) and electroencephalogram changes. The results of animal behavior experiments showed that NEMP exposure induced rats to an anxious state, as evidenced by reduced dwelling time and traveling distance in open arms in EPM experiment, which could be explained by a greater reluctance of rats to venture into risky activities. In addition, the results of OPT experiments also showed that NEMP exposure led to less autonomous and exploratory behaviors in rats. Importantly, our results show that H_2_ has a protective effect against anxiety-like behavior in rats induced by NEMP exposure. The results of animal behavioral experiments with EMR are usually confusing and contradictory, mainly because the effects of EMR on brains are related to its type, intensity, frequency, and duration of exposure. Different parameters may lead to opposite conclusions. It has been demonstrated that long-term exposure to very low frequency electromagnetic fields has pro-anxiety and oxidative stress-inducing effects in rats (20). Zhang et al. (2) studied the behavioral effects of 1.8 GHz radiofrequency fields on mice and found a similar phenomenon. In contrast, Qubty et al. (21) found no difference in anxiety-like behavior in EPM experiment of mice exposed to radiofrequency EMR. It is worth mentioning that in our previous study, we found that the negative effects of EMR exposure decayed quickly over time (11), for which only the very acute (one day after exposure) underlying biomolecular changes were determined by using the transcriptomic, metabolomic and behavioral means to dissect the potential molecular alterations induced by NEMP and/or H_2_ exposure.

The hippocampus is the place where human conceptions of space and time are intertwined with memory (22). And the hippocampus is thought to be a vulnerable brain region to EMR induced injury (23). Exposure to 900/1,800 MHz radio frequency electromagnetic waves has been reported to cause oxidative damage to the hippocampus in mice (24). The amygdala is a crucial area of the brain that regulates motivation behavior and regulate switches between exploratory and non-exploratory defensive state (25). The results of histopathological experiments showed that significant changes had taken place in hippocampal and amygdaloid neurons following NEMP exposure in rats: cell swelling, pronounced cellular gaps, morphological changes (from round and oval to triangular), deepening cell staining, and blurring of the nucleus and cytoplasm. Interestingly, molecular hydrogen could significantly attenuate these damages to neurons. These histopathological results provided critical evidence to explain the anxiety-like changes in rats after NEMP exposure in animal behavioral experiments, as well as the alleviation of anxiety-like changes after molecular hydrogen treatment. In the present study, H_2_ counteracted the histological changes in most neurons in whole hippocampal and amygdala after NEMP exposure. The results demonstrated that molecular hydrogen might work without regional specificity.

Microtubular structure plays a key role in myelination, neuronal cytoskeletal junction, and cell-matrix attachment and has a dramatic effect on brain stiffness (26). The microtubular structure is also susceptible to EMR, and 915 MHz radiofrequency EMR can disrupt microtubule structures and perturb cell growth (27). Our transcriptomics data showed that NEMP exposure has the most significant effect on microtubular structures, with biological process terms including axoneme assembly, microtubule bundle formation, microtubule cytoskeleton organization, microtubule-based process, etc. This may be attributed to the possibility that NEMP exposure affects cytoskeletal proteins within neurons and glia, and explain the neuronal damage caused by NEMP exposure. In addition, this will be one of our subsequent research directions, and further research will be conducted later using techniques such as electron microscopy.

Molecular hydrogen is a new type of antioxidant with potent free radical scavenging ability, and its exertion of biological effects may also result from its extensive influence on metabolic pathways (28). Consistently, metabolomics results showed that H2 performed as a potent modulator in regulating metabolic pathways, mainly impacting molecules relating with neuroactive ligand-receptor interaction, synaptic vesicle cycle, synapse, cAMP signaling pathway and drug addiction et al. In contrast, the NEMP exposure-induced disturbance was only enriched to an irrelevant pathway, probably because the effect of NEMP exposure on brains was not obvious in metabolic pathways.

GSH plays a vital role in the neuronal antioxidant defense system and in maintenance of neuronal redox homeostasis. Thus, the consumption of GSH in the brains is a common contributor to patients with neurodegenerative diseases such as Alzheimer's disease and Parkinson's disease (29). GSSG is the oxidative state of GSH, and GSH/GSSG is often used as a sensitive indicator to evaluate the intracellular antioxidant capacity (14). Although the differences were not statistically significant due to excessive intragroup dispersion, the elevation of GSSG caused by NEMP exposure and the decline of GSSG induced by molecular hydrogen treatment could be easily observed. For NADPH, which is the hydrogen donor in the process of reducing GSSG to GSH, its oxidized product NADP is also increased after NEMP exposure and decreased after H_2_ treatment. Additionally, H_2_ also significantly reduced the expression of gpx3, which is a key enzyme for oxidizing GSH to GSSG. These results suggest that NEMP exposure interferes with the GSH reduction system and causes oxidative stress to neurons, which in turn leads to the onset of neuronal damage. Our work is consistent with a study which demonstrated that electromagnetic waves emitted by cell phones reduce glutathione levels in the brains of mice (30).

The present study only tentatively validated the protective effect of H_2_ on brain injury induced by NEMP exposure and explored the potential mechanisms, with many limitations and controversies unsettled. Brain damage induced by NEMP exposure also needs more efficient and instant markers, such as TUNEL staining. Concerning the effect of NEMP on microtubule structure, we only made a preliminary step in exploring the mechanism of molecular hydrogen treatment, and more basic experiments are needed to corroborate our transcriptomic results subsequently. Most importantly, the present study identifies H_2_ as an efficient and potent protectant against NEMP exposure-induced brain injuries, paving the way for future translational study.

## Conclusions

Overall, the present study demonstrated the protective effect of H_2_ against brain damage induced by NEMP exposure in rats. Mechanistically, NEMP exposure mainly disrupts microtubule structure and H_2_ exerts protective effects by modulating metabolic pathways, among which the most affected is glutathione metabolic pathway.

## Data availability statement

The original contributions presented in the study are included in the article/Supplementary material, further inquiries can be directed to the corresponding authors.

## Ethics statement

The animal study was reviewed and approved by Naval Medical University Animal Welfare and Ethics Committee.

## Author contributions

LM contributed to experimental operation, exposure process, first draft writing, and experimental design of the study. J-MG contributed to experimental design, exposure process, and manuscript revision. ST contributed to data analysis and animal experiments. H-LZ contributed to statistical analysis of the study. QS contributed to the data analysis and manuscript revision. X-GH contributed to the study design and bioinformatic analysis. H-LY, J-YW, and J-WW contributed to the animal feeding, histological analysis, hydrogen-rich water preparation and concentration measurement, and data statistics.
