# Supplementary material for: Transcriptomic and metabolomic studies on the protective effect of molecular hydrogen against nuclear electromagnetic pulse-induced brain damage
Source: Front Public Health. 2023 Feb 1;11:1103022. doi: 10.3389/fpubh.2023.1103022 (PMC9929151; doi:10.3389/fpubh.2023.1103022)
Supplement: Supplementary file 2 [file Data_Sheet_1.DOCX]

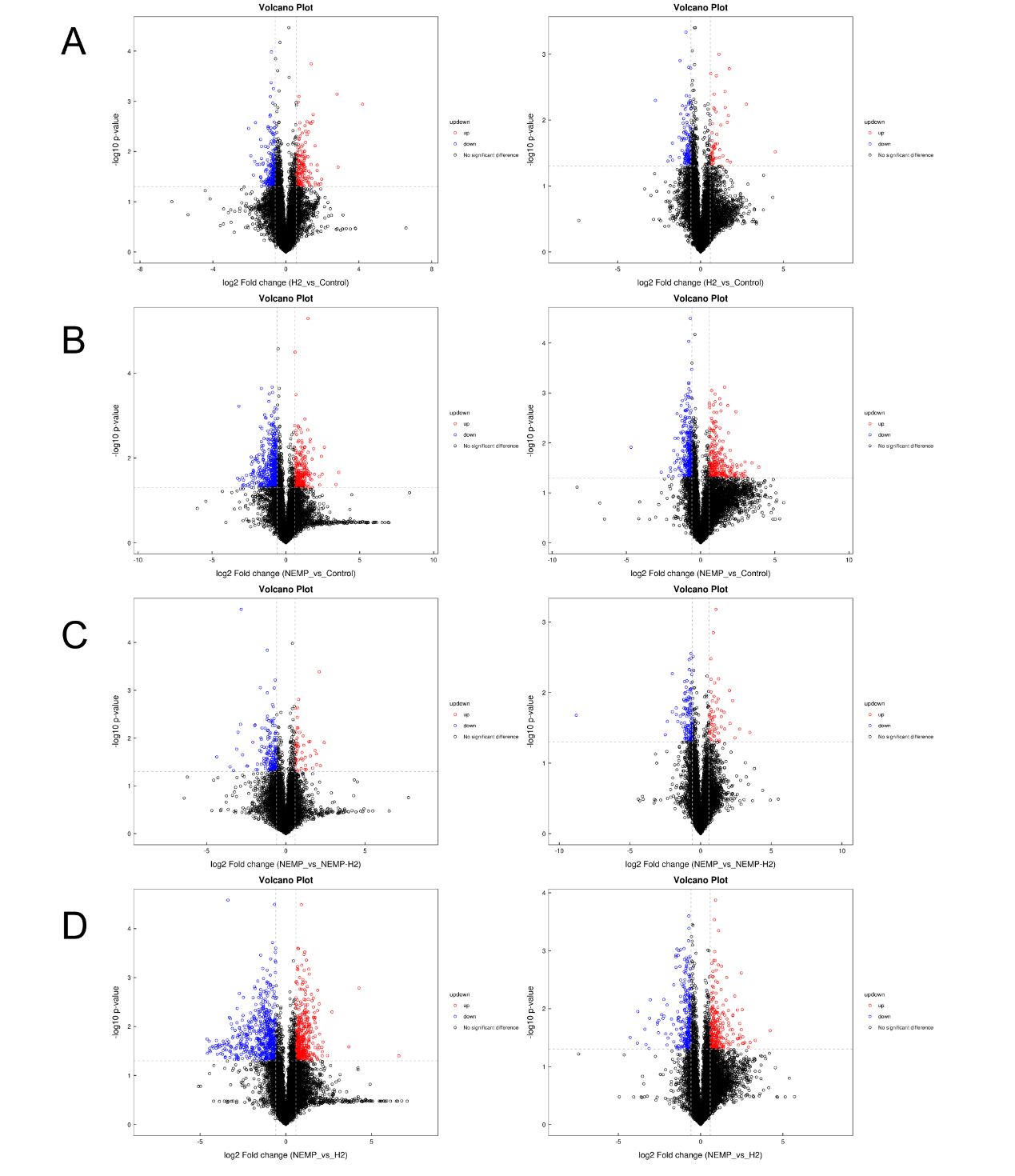


Figure S1 Volcano plots of the differential expression profile of metabolites. (A) The H_2_ group vs. the control group (positive and negative ion mode). (B) The NEMP group vs. the control group (positive and negative ion mode). (C) The NEMP group vs. the NEMP+ H_2_ group (positive and negative ion mode). (D) The NEMP group vs. the H_2_ group (positive and negative ion mode). The horizontal coordinate is the log2 value of the differential expression multiplier, and the vertical coordinate is the log10 value of the significant P value. Metabolites meeting foldchange>1.5 and p value <0.05 are shown in red, and metabolites that meet foldchange <0.67 and p value <0.05 are shown in blue. No significantly different metabolites are shown in black.


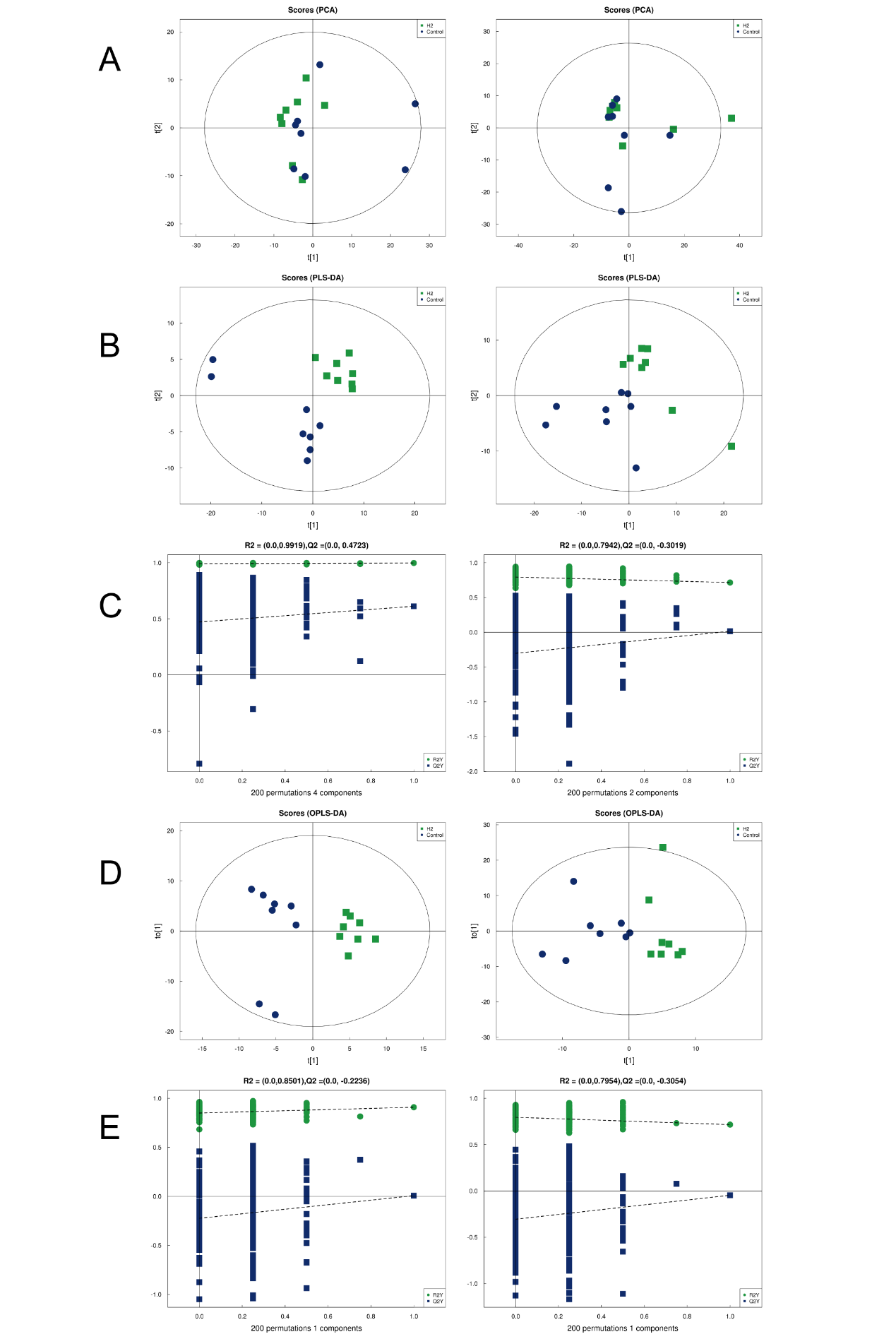


Figure S2 Multivariate statistical analysis of DMs between the H_2_ group and the control group. (A) PCA score graph. (B) PLS-DA score graph. (C) PLS-DA permutation test. (D) OPLS-DA score graph. (E) OPLS-DA permutation test. In the score graphs, t[1] represents principal component 1, t[2] represents principal component 2, and the ellipse represents the 95% confidence interval. The dots of the same color indicate the individual biological replicates within the group. The distribution status of the points reflects the degree of variation between and within groups.


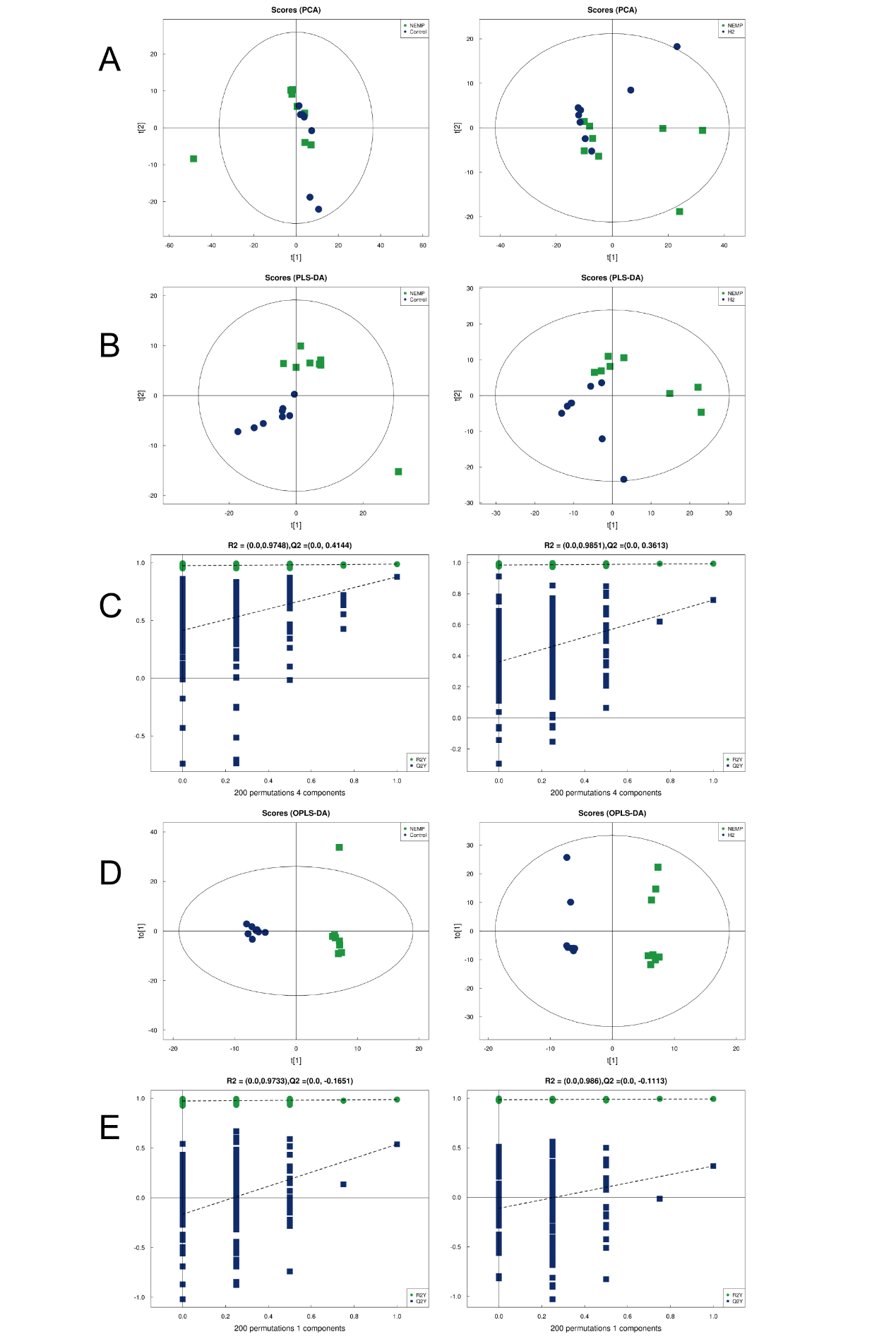


Figure S3 Multivariate statistical analysis of DMs between the NEMP group and the control group. (A) PCA score graph. (B) PLS-DA score graph. (C) PLS-DA permutation test. (D) OPLS-DA score graph. (E) OPLS-DA permutation test. In the score graphs, t[1] represents principal component 1, t[2] represents principal component 2, and the ellipse represents the 95% confidence interval. The dots of the same color indicate the individual biological replicates within the group. The distribution status of the points reflects the degree of variation between and within groups.


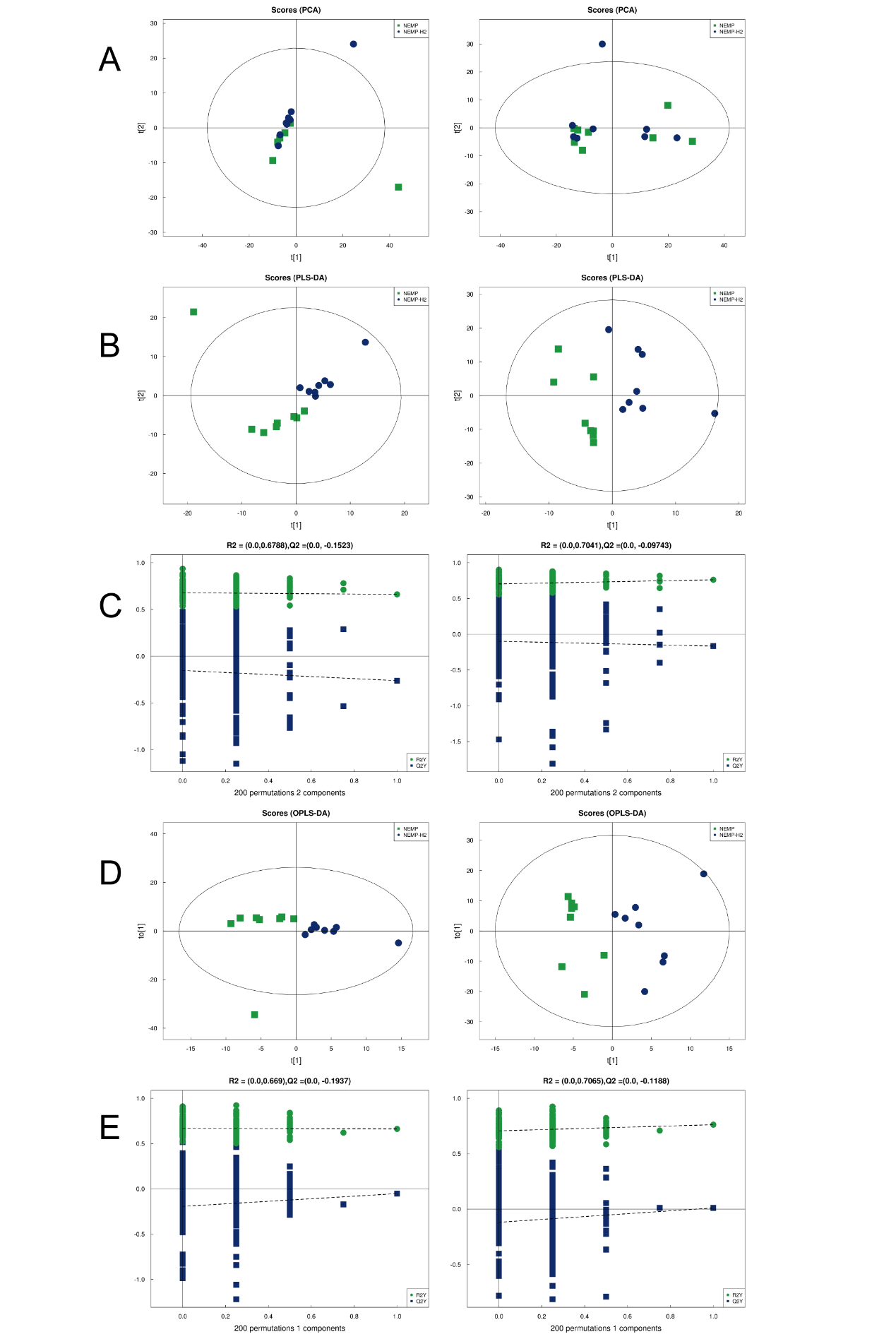


Figure S4 Multivariate statistical analysis of DMs between the NEMP+H_2_ group and the NEMP group. (A) PCA score graph. (B) PLS-DA score graph. (C) PLS-DA permutation test. (D) OPLS-DA score graph. (E) OPLS-DA permutation test. In the score graphs, t[1] represents principal component 1, t[2] represents principal component 2, and the ellipse represents the 95% confidence interval. The dots of the same color indicate the individual biological replicates within the group. The distribution status of the points reflects the degree of variation between and within groups.


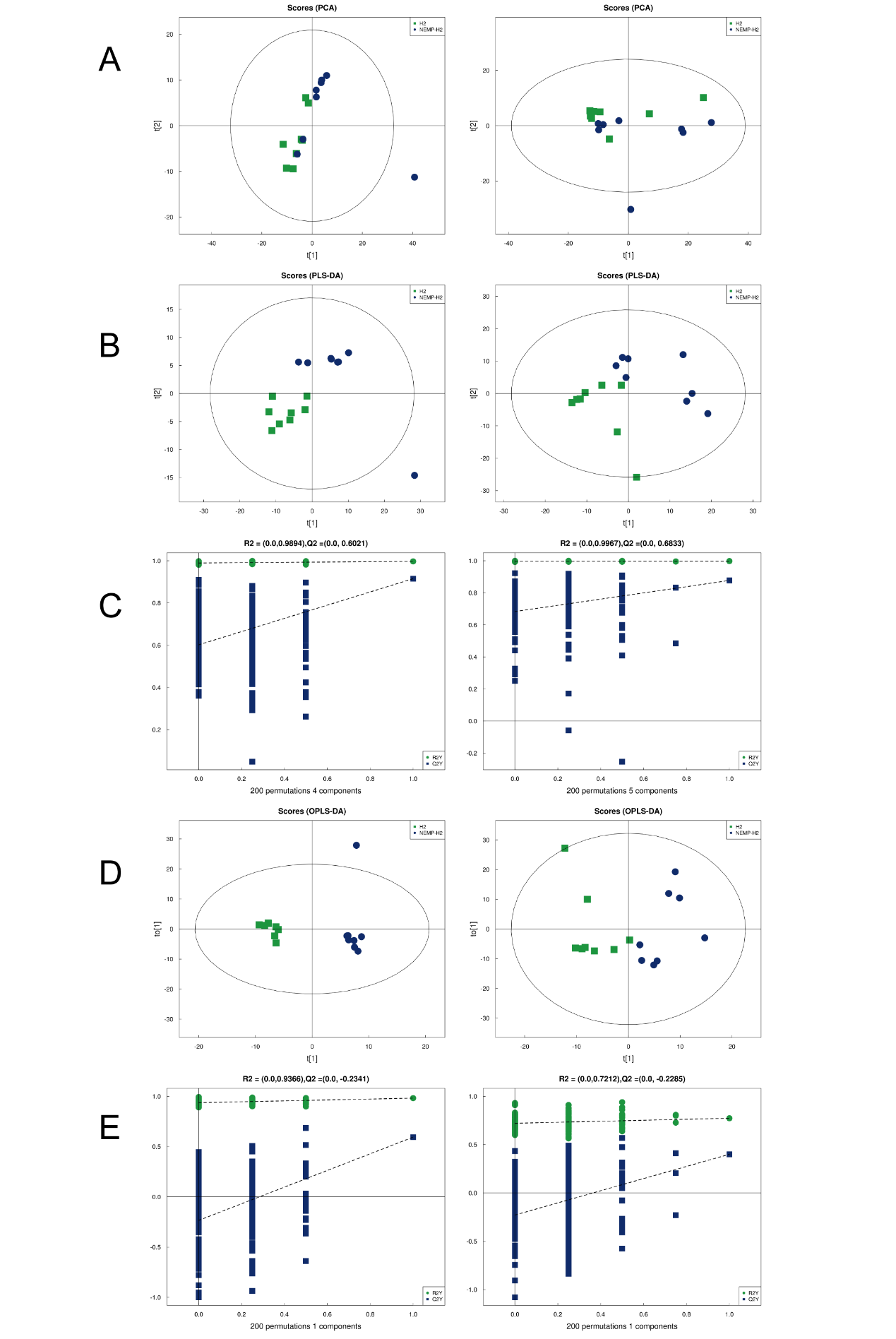


Figure S5 Multivariate statistical analysis of DMs between the NEMP+H_2_ group and the H_2_ group. (A) PCA score graph. (B) PLS-DA score graph. (C) PLS-DA permutation test. (D) OPLS-DA score graph. (E) OPLS-DA permutation test. In the score graphs, t[1] represents principal component 1, t[2] represents principal component 2, and the ellipse represents the 95% confidence interval. The dots of the same color indicate the individual biological replicates within the group. The distribution status of the points reflects the degree of variation between and within groups.


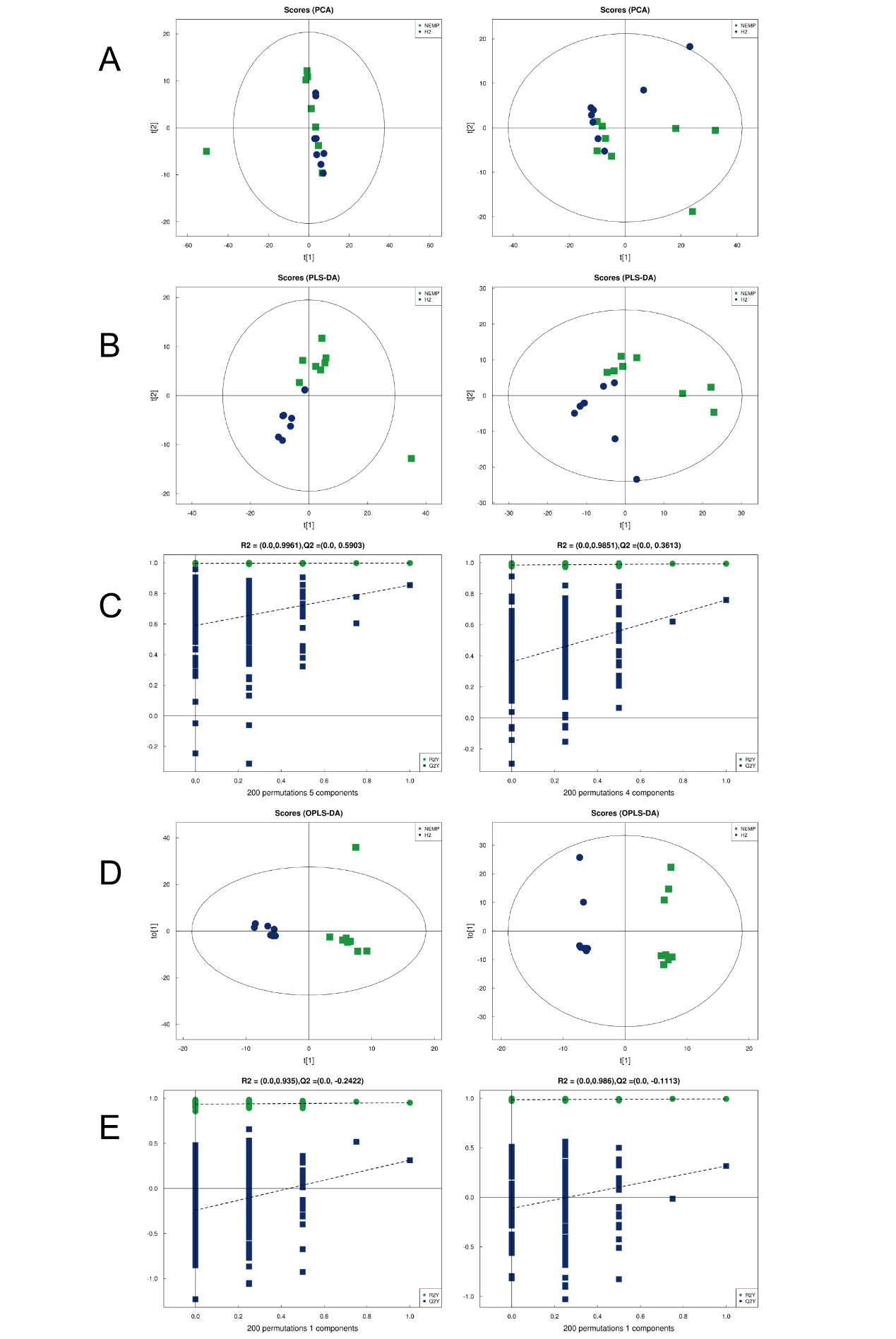


Figure S6 Multivariate statistical analysis of DMs between the NEMP group and the H_2_ group. (A) PCA score graph. (B) PLS-DA score graph. (C) PLS-DA permutation test. (D) OPLS-DA score graph. (E) OPLS-DA permutation test. In the score graphs, t[1] represents principal component 1, t[2] represents principal component 2, and the ellipse represents the 95% confidence interval. The dots of the same color indicate the individual biological replicates within the group. The distribution status of the points reflects the degree of variation between and within groups.


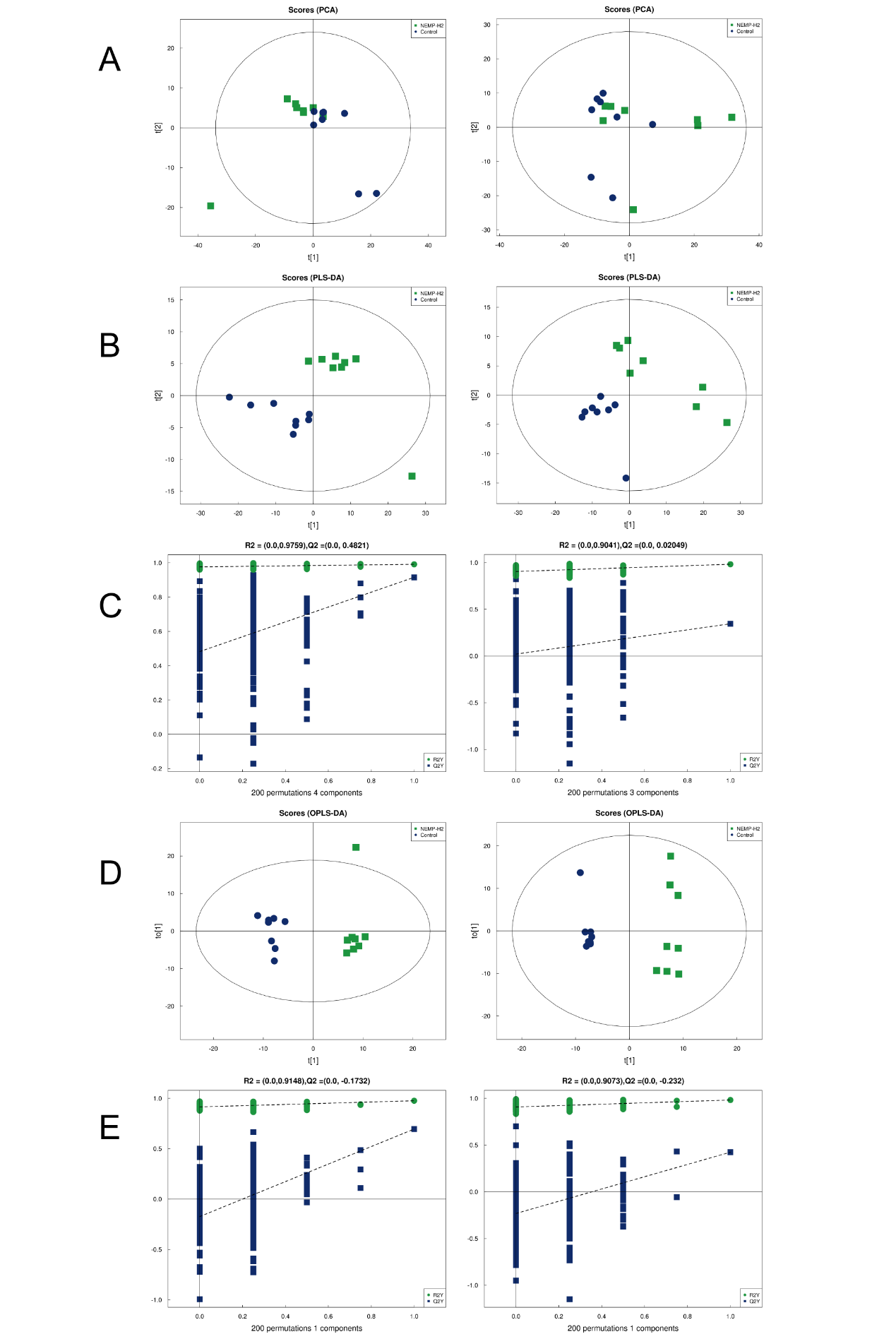


Figure S7 Multivariate statistical analysis of DMs between the NEMP+H_2_ group and the control group. (A) PCA score graph. (B) PLS-DA score graph. (C) PLS-DA permutation test. (D) OPLS-DA score graph. (E) OPLS-DA permutation test. In the score graphs, t[1] represents principal component 1, t[2] represents principal component 2, and the ellipse represents the 95% confidence interval. The dots of the same color indicate the individual biological replicates within the group. The distribution status of the points reflects the degree of variation between and within groups.


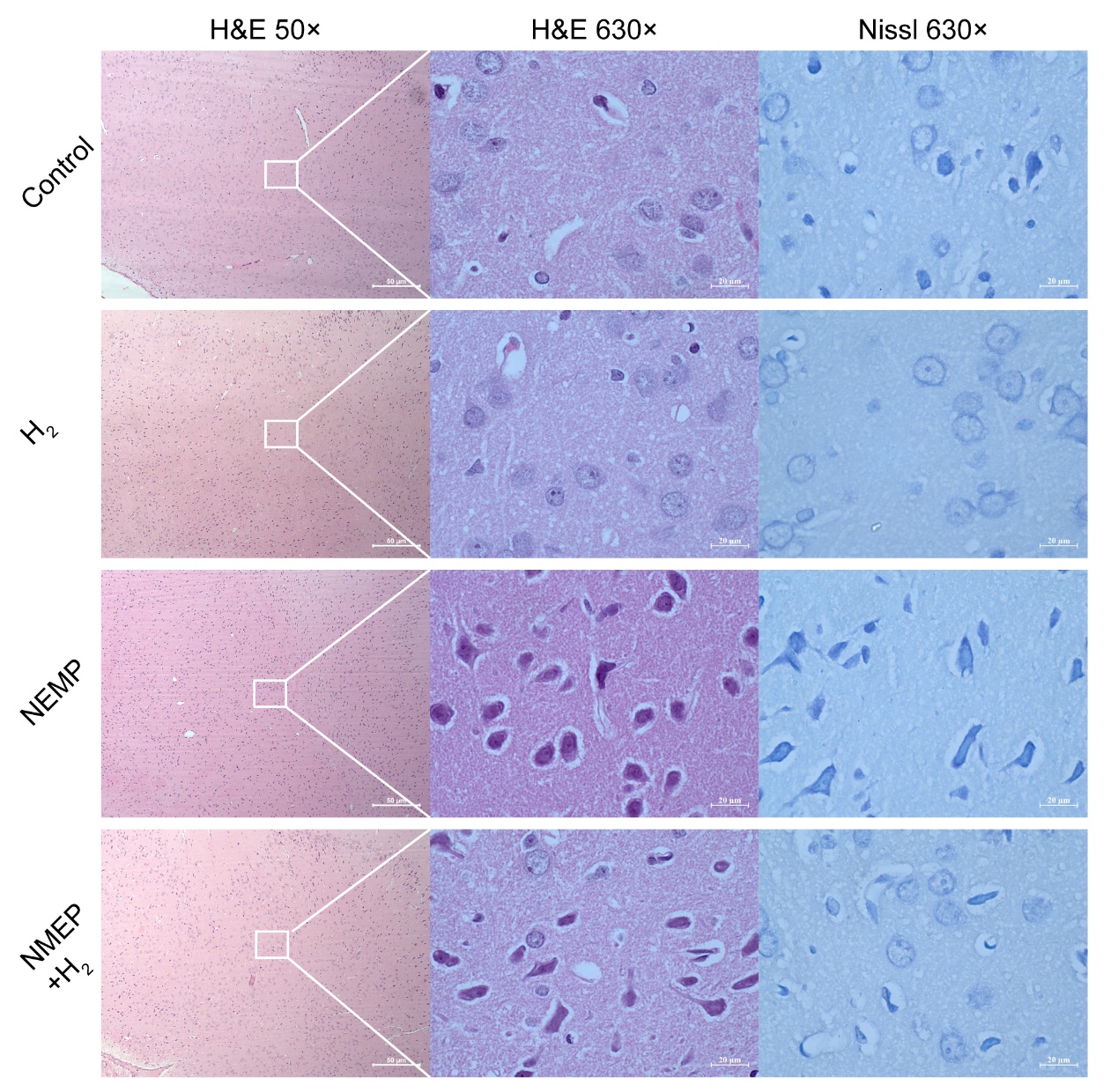


Figure S8 Representative micrographs of the amygdala in each group (H&E and Nissl staining). N=4


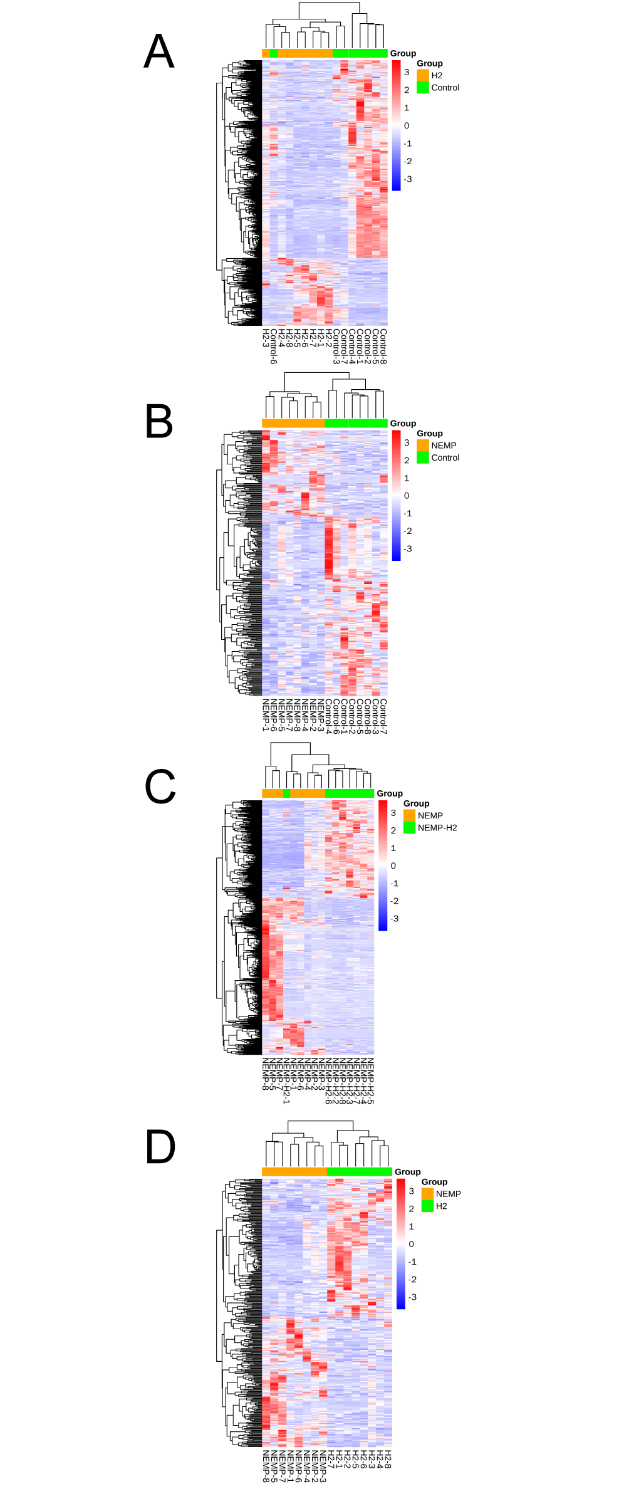


Figure S9 Heatmap of the differential expression profile of genes. (A) The H_2_ group vs. the control group. (B) The NEMP group vs. the control group. (C) The NEMP group vs. the NEMP+ H_2_ group. (D) The NEMP group vs. the H_2_ group. Each column represents one sample, and each row represents one gene. Red indicates up-regulation and blue indicates down-regulation. The upper side shows the tree diagram of the sample clustering. The closer the branches of two samples are to each other, the closer the expression pattern of all differential genes in these two samples. The left side shows the tree diagram of gene clustering. The closer the two gene branches are to each other, the closer their expressions are.


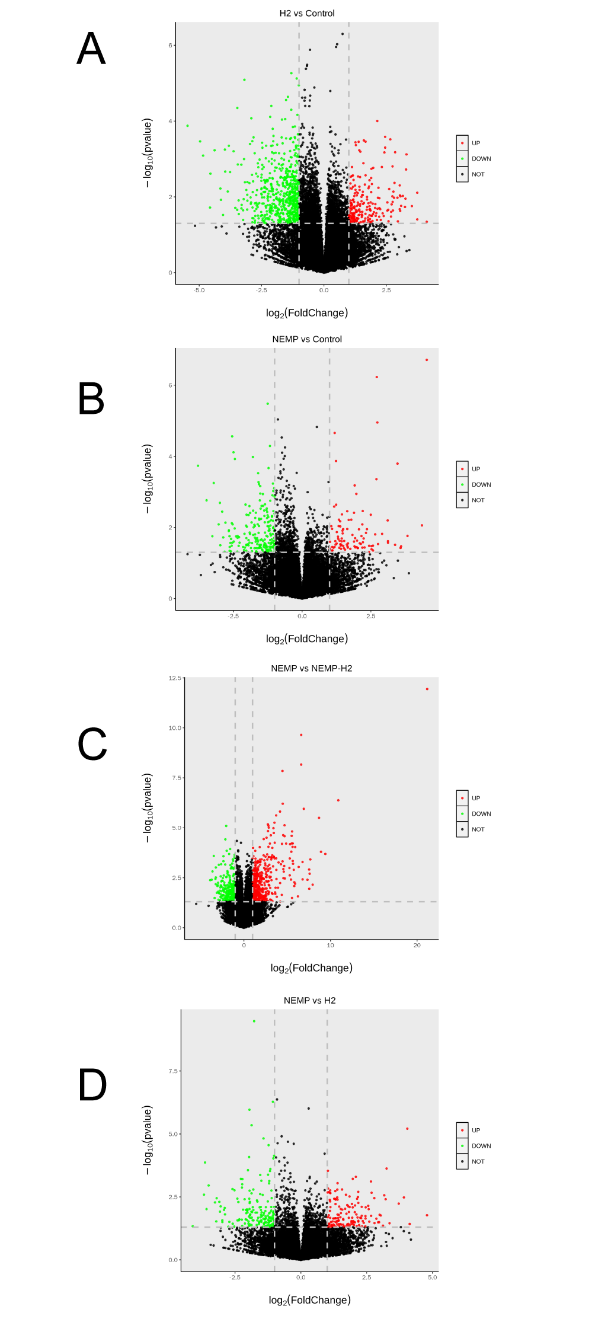


Figure S10 Volcano plot of the differential expression profile. (A) The H_2_ group vs. the control group. (B) The NEMP group vs. the control group. (C) The NEMP group vs. the NEMP+ H_2_ group. (D) The NEMP group vs. the H_2_ group. Each point in the differential expression volcano plot represents a gene, the abscissa represents the logarithm of the difference in the expression level of a gene between two samples, and the ordinate represents the negative logarithm of the P-value. The green dots represent down-regulated DEGs, the red dots represent up-regulated DEGs, and the black dots represent non-DEGs.
